# Multi-Scale Multi-Indicator Assessment of Material Recovery Pathways: The Case of the United States Flat Glass Sector

**DOI:** 10.1021/acs.est.6c01727

**Published:** 2026-10-23

**Authors:** Julien Walzberg, Nivedita Biyani, Shubhankar Upasani, Mansi Sharma, Liz Wachs, Rebecca J. Hanes, Tapajyoti Ghosh, Andre Avelino, Annika Eberle, Alberta Carpenter

**Affiliations:** 53405 National Laboratory of the Rockies, 15013 Denver West Parkway, Golden, Colorado 80401, United States

**Keywords:** input output modeling, life cycle assessment, material recovery, flat glass, material flows, local manufacturing

## Abstract

Material recovery and circularity analysis offers critical insights for understanding future scenarios and informing strategic decisions toward resource use. However, existing approaches often lack the granularity and breadth needed to capture supply chain dynamics and economy-wide impacts simultaneously. We introduce a novel quantitative framework—Supply Chain Operations Research through Physical Input-Output (SCORPIO)—that addresses this gap by enabling multi-scale assessment of material recovery strategies across both supply chain and macroeconomic levels. To demonstrate SCORPIO’s capabilities, we apply it to flat glass flows in the United States, examining waste valorization practices, including open- and closed-loop recycling. By assessing economic, workforce, and environmental indicators, SCORPIO reveals the potential outcomes and trade-offs of different waste valorization solutions in a holistic manner. Results show that both open-loop and closed-loop recycling of glass from utility solar and commercial buildings have the potential for positive economic impacts at the supply chain and economy level. However, the effect on environmental impacts was less certain, with open-loop recycling generally performing better than closed-loop pathways. This case study illustrates how the SCORPIO framework can support stakeholders navigating the transition toward increased material recovery, offering a methodological foundation applicable to other material systems facing environmental and resource challenges.

## Graphical abstract

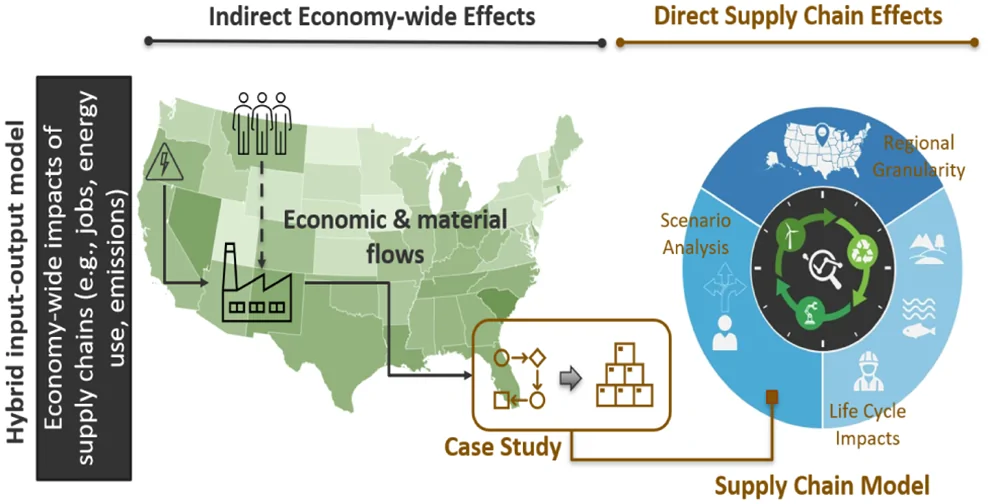

## 1 Introduction

### 1.1 Research Gap and Research Objectives

The circular economy paradigm represents a transformative shift from traditional linear economic models to systems that prioritize resource efficiency, waste minimization, and sustainability. Its objective is to keep the value of materials and products within the economy by *narrowing* (e.g., through product designs with lower material intensity), *slowing* (e.g., by extending product lifetimes), *regenerating* (e.g., by using resources that are renewable), and *closing* (e.g., through recycling) material flows.^1^ This objective is operationalized through frameworks such as the zero-waste hierarchy, where refusing, rethinking, and reducing are the first points of intervention, followed by reuse, repair, refurbish, remanufacturing, and repurposing, before finally arriving at recycling and recovering.^2^ This research focused on recycling, assuming prior strategies have been considered and implemented, considering that they are highest-order interventions for circularity. The scope of this study is explicitly recycling pathways. As a subset of the circular economy, material recovery has the potential to ease supply chain constraints (especially for critical minerals and materials) by reducing primary demand and providing domestic sources while bolstering economic competitiveness through the creation of new domestic industries processing secondary materials and providing waste valorization services. Material recovery aims to collect waste materials and give them a second life through reuse, recycling, and regeneration. It could improve the efficiency of resource use (energy, materials, water, and land) and create value within the economy.^3^ On the other hand, some material recovery practices can be costly, less profitable, or difficult to implement because of conflicting business models and supply chain uncertainty. Trade-offs may also appear between different economic, social, and environmental indicators; hence the need to ensure that the benefits of material recovery pathways are not outweighed by their negative consequences (e.g., displacement of jobs from one region to another, shifting environmental burdens, and economic losses for economic sectors producing virgin materials).

Countries such as the United Kingdom, and other European countries, China, and Canada are increasingly using material recovery strategies to strengthen their supply chains and decouple economic activities from environmental impacts.^4–6^ However, adequate analysis frameworks for modeling their consequences at multiple scales are lacking. Considering material recovery at different levels of analysis is crucial to understand unintended consequences such as impact displacements,^7^ which may limit its value proposition. For instance, Lonca, Lesage, Majeau–Bettez, Bernard, and Margni^8^ showed that increased closed-loop recycling of polyethylene terephthalate (PET) bottles is environmentally beneficial when the scope of the analysis is at the product level, but not when the scope is at the whole PET market level. Indeed, the study finds that increasing closed-loop recycling (focusing on the product, PET bottles) to the detriment of open-loop recycling worsens the environmental impacts of the United States (U.S.) plastic system (i.e., when conducting the analysis at a larger scale).

Modeling material recovery at different scales involves developing frameworks that simulate the dynamic interactions among economic activities, environmental impacts, and resource flows under circularity principles. Pathways for achieving widespread material recovery in the United States remain unclear; and while benefits are expected, they have not been adequately quantified at meso- and macrolevels. It is therefore important to conduct analysis to evaluate and compare material recovery pathways, understand system interactions, quantify material recovery benefits and trade-offs, and anticipate barriers to technology uptake.

Many models have been developed to understand opportunities for product/material recovery and secondary use at different scales.^9,10^ However, those models often lack the multi-scale analysis capabilities that are necessary to assess the trade-offs of material recovery between economic sectors or between socio-economic and environmental objectives. Gillingham et al.^11^ arrive at the same conclusions for the industrial sector: more research combining detailed industry-level analyses and macro-level analyses is needed to capture broader consequences such as rebound effects and impact leakages. As such, this study builds on previous work by integrating existing models into a multi-scale analysis framework for material recovery strategies.

Similarly, several methodologies measuring performance across different indicators have been applied to study circularity.^10^ However, these methodologies often do not perform assessments at multiple scales. For instance, the Ellen MacArthur Foundation’s material circularity index is well adapted to product-level or company-level circularity assessment but lacks considerations of the larger economy, such as required infrastructure and inter-sector trade-offs.^12^ Conversely, the European Union’s Circular Economy Monitoring Framework defines circularity-related indicators in four areas (production and consumption, waste management, secondary raw materials, competitiveness and innovation, and global sustainability and resilience) that are well adapted to assess circularity at the macro level but are difficult to apply to specific supply chains.^13,14^ Integrated assessment models (IAMs) provide a large view of socio-economic and natural systems but ignore material cycles, leading to inaccuracy in evaluating the impacts of technologies and their potential for increased circularity.^15^ Combining IAMs with industrial ecology tools such as life cycle assessment (LCA) and material flow analysis (MFA) can solve those issues to some extent,^15^ as demonstrated in several studies.^16–19^ Pehl et al.,^16^ for instance, use a prospective LCA model to calculate indirect embodied impacts of energy technologies and add them to the direct impacts calculated by an IAM. In another study, the IMAGE integrated assessment model is combined with a dynamic stock and flow model to evaluate future demand for copper, neodymium, and tantalum.^17^ However, those frameworks still suffer from a lack of sectoral and supply chain details.^20^

The closest methodology to perform environmental assessments at both the supply chain (meso) and economy-wide (macro) levels is hybrid LCA. This methodology allows researchers to model the environmental impacts at both the product and economy-wide levels, taking advantage of the precision of LCA and the comprehensiveness of environmentally extended input-output (EEIO) analysis.^21^ However, there is no single, harmonized hybrid LCA method, and the methodology focuses on environmental impacts^21,22^ without considering economic impacts. Moreover, only a few studies have used hybrid LCA to study material recovery systems (see, for instance, Gueddari-Aourir et al.^23^), and none have focused on the U.S. economy. Given that material recovery systems vary case-by-case—depending on materials, geography, and time—it is crucial to develop adequate methodologies that can evaluate the environmental, social, and economic costs and benefits of increased material recovery within the U.S. economy.

Hence, from the reviewed literature, we observe that there is currently no comprehensive approach that enables performing circularity assessments at multiple scales while accounting for economic, workforce, and material-flow impacts, in addition to environmental ones. To address this gap, this article aims to:

- Propose a novel approach to assess material recovery at multiple scales and through the lens of multiple indicators based on a detailed supply chain model and economy-wide input-output tables expressed in monetary and physical units.
- Apply the proposed approach on a case study of flat glass recovery in the U.S. to answer the following research questions:
  What processing cost thresholds lead to an increase in flat glass material recovery? How do these thresholds differ across material recovery pathways?
  What are the economic and environmental impacts associated with closed-loop material recovery pathways? How do they compare with open-loop material recovery pathways? How do the economic and environmental impacts of material recovery supply chains compare to the baseline supply chain mostly relying on landfilling?
  What is the impact on net jobs with the development of flat glass reverse supply chains? Across the entire U.S. economy, is the net job change associated with a particular material recovery scenario positive or negative? What are the broader economic implications of material recovery? How do they modify the structure of material flows within the economy?

### 1.2 Current State of Glass Recovery

Global demand for glass is about 150 million metric tonnes (Mt), of which 48% is container glass used mainly for beverages, 42% is flat glass used primarily for windows in buildings and cars, 5% is tableware, and 6% is from other types of products such as glass fibers.^24^ Most of the glass is produced using circa 15% soda, 10% lime, and 75% silica.^25^ Glass manufacturing is estimated to consume 800 petajoule of energy worldwide in 2007—with about a quarter from the U.S. glass industry.^26^ Hence, the sector is among the most energy-intensive representing about 1.6% of the 19,237 trillion British thermal units of primary energy consumed by the U.S. manufacturing sectors in 2010.^27^ However, reuse and recycling rates^1^ remain low. Increasing material recovery for glass manufacturing could save raw materials, decrease manufacturing costs, reduce energy use, and reduce environmental impacts.^28^ In the U.S., it is estimated that 31% of container glass is recycled^29^ either into new containers or downcycled into other products, such as glass fibers or construction aggregates, and less than 5% of flat glass is downcycled into construction aggregates, with the rest being landfilled.^30^ The reuse rate for container glass is also low: 4% in the EU and even lower in other countries, with the exception of Canada, where the reuse rate is 68% (as many glass bottles are handled through deposit-return systems).^31^

Low recycling and reuse rates represent missed economic and environmental opportunities. Every 10% of glass raw materials replaced with recycled glass cullet, reduces energy consumption in the melting process by 3%,^28^ and Westbroek et al.,^25^ showed that using cullet in glass manufacturing lowers costs associated with raw materials and energy use.^32^ While total manufacturing costs depend on cullet prices, a recent study has found that recycling could generate economic benefits of $11.6 per tonne of flat glass for manufacturers.^33^

Other material recovery strategies represent more limited opportunities.^25^ For instance, the remelting of internal and pre-consumer cullet is already common practice, leaving little room for improvement. Extending the lifetimes of cars and buildings could reduce the demand for glass, but would have only a small overall impact, since those products already have long lifetimes. Finally, re-designing glass products could also reduce demand and impacts, but it is uncertain how those changes might affect the glass supply chain material and energy flows. Hart, Selkowitz, and Curcija,^34^ for instance, have shown that thin triple-paned windows (a new design destined to replace conventional double-paned windows) could save up to 16% of residential buildings’ energy consumption for a small increase in material use and embodied energy.^35^

Glass products present varying sensitivity to process conditions. The more constrained the process, the higher the costs of production and value of the glass product. As a result, upcycling glass is not possible since the introduction of cullet—generally with higher levels of contaminants than virgin materials—would cause significant production losses. Indeed, contaminants lead to several issues, such as the buildup of ceramic or metal inclusions within the glass, melting disturbances caused by limited heat transfer, and downward drilling of melts of metals present in the cullet.^36^ Since upcycling strategies are technically limited, this study focuses mainly on two material recovery strategies for flat glass: closed-loop and open-loop recycling. Moreover, since container glass is currently recycled in the U.S. (albeit at a moderate recycling rate) but flat glass is not, the rest of the article will mainly discuss material recovery of flat glass.

## 2 Materials and Methods

We combine three previously developed supply chain modeling tools—the Circular Economy Life Cycle Assessment and Visualization (CELAVI), the Physical Input-Output for Circularity Analysis (PIOCA), and the Environmentally extended Multi-regional Projection of Lifecycle and Occupational energy futures (EMPLOY) into the broader Supply Chain Operations Research through Physical Input-Output (SCORPIO) framework to explore the socio-economic and environmental impacts and trade-offs of material recovery pathways at different scales. This section first succinctly describes the CELAVI, PIOCA, and EMPLOY frameworks. Next, we detail how those models are combined through soft linkages to form SCORPIO. Finally, we discuss the data used to model glass flows in the U.S. for a baseline scenario and a range of material recovery scenarios.

### 2.1 Building the Multi-Scale, Multi-Indicator SCORPIO Framework

CELAVI is a supply chain simulation model that integrates a network model of supply chain facility locations and processing steps, with a discrete-event simulation to capture technology component-level material flows and dynamic LCA to quantify spatially and temporally explicit environmental impacts. CELAVI uses a high-resolution, cost-minimizing decision model to determine how technology components are processed at end-of-life and simulates material flows over time as a function of exogeneous demand for new technology components and the endogenous, dynamic supply chain structure. CELAVI was built to be technology- and material-agnostic and has previously been applied to glass-fiber-reinforced polymer wind turbine blades. Complete methodological details for CELAVI are available in refs 37,38 , and methodological details on how sensitivity analyses are run in CELAVI are available in ref 39 CELAVI is implemented in Python, and the model version used in this study is publicly available at GitHub.

Whereas CELAVI captures meso-scale material flows, costs, and impacts, EMPLOY and PIOCA investigate macro-scale economic and physical material flows and impacts. EMPLOY is an environmentally-extended input-output model designed to assess the economy-wide effects of expanding U.S. economic sectors across multiple dimensions: economic (jobs, value-added, economic activity), environmental (releases to air, soil, and water), and workforce (occupations). The model combines the non-linear input-output framework from Guerra and Sancho^40^ with the FERMIO model from Wiebe, Bjelle, Többen, and Wood,^41^ allowing it to forecast impacts in the U.S. economy up to 2050 at the state-level. EMPLOY’s environmental and LCA accounts were estimated following the methodology from Yang et al.^42^ and reflect conditions in 2017.^43^

PIOCA creates a physical (mass) representation of the U.S. economy, which can be used for visualization and analysis. The PIOCA codebase derives a price vector primarily from trade data and statistics from the U.S. Geological Survey (USGS).^44^ The codebase uses IO tables from the U.S. Bureau of Economic Analysis (BEA, at the summary level, i.e., for 71 economic sectors for SCORPIO, although the detail level can also be used), and all economic flows within the BEA tables (except for services) are converted into physical flows. For this work, a full table was derived based on the 2012 economy. As constant prices are assumed in EMPLOY, the 2050 conversion is based off the same price vector, with the exception of the sectors involved in material recovery (i.e., flat glass production and recycling), whose prices are recalculated due to changing input composition. Material recovery strategies can modify physical flows in the economy without changing monetary flows (or at least not in the same proportions), so PIOCA is theoretically better adapted to material recovery analysis than monetary-based IO. However—contrary to EMPLOY—PIOCA does not compute economic metrics and does not have prospective analysis capabilities. For this reason, the combined method is used for this work. More information on the three models used within SCORPIO is available in the Supplementary Information file (Section 1).

To connect supply chain and economy-wide modeling (Figure 1 and Supplementary Figure S1), SCORPIO first disaggregates the economic sector of interest (defined at the 3-digit North American Industry Classification System (NAICS) code level) into an array of detailed sectors in EMPLOY (defined at the 6-digit NAICS code level). Input and output commodities of the sector were also adapted to fit the newly created detailed sectors. Finally, entirely new sectors are created in EMPLOY to represent material recovery pathways. Section 3.2 details how this was achieved for the flat glass case study.

**Figure 1:**
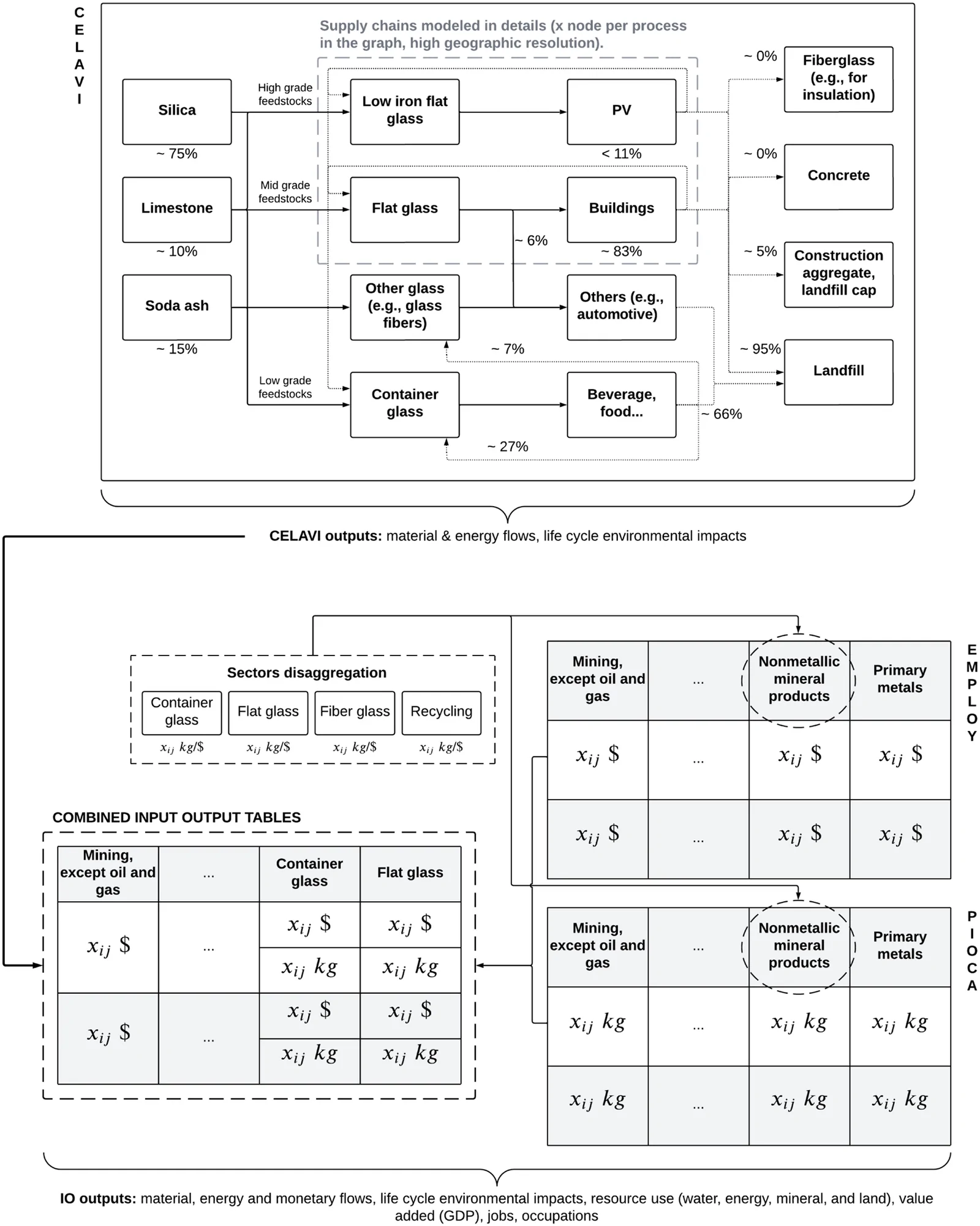
SCORPIO framework overview.

Next, we align PIOCA sectors to those of EMPLOY, yielding similar sized technical coefficient matrices and final output vectors. The new sectors added in EMPLOY are also added in PIOCA, using its price vector to convert any new monetary flows into physical ones. Once both IO models are aligned, a Python script converts any new EMPLOY results into its physical counterparts in PIOCA. CELAVI material flows in the different landfilling and material recovery pathways of the supply chain are used as the IO models’ inputs. Section 1 of the Supplementary Information provides more details on how the models are connected.

### 2.2 Data Collection and Material Recovery Pathways

For a given technology or product, CELAVI simulates supply chain structures and material flows over time in response to the availability of material recovery options at a technology’s end-of-life and the relative costs of landfilling and material recovery pathways (Supplementary Figure S2). Data requirements for CELAVI include geographic locations for all facilities in the supply chain superstructure; spatio-temporally explicit historic and projected demand for all technologies being simulated; process costs for every supply chain processing step (there may be multiple processing steps at a single supply chain facility) and transportation type; and material losses and waste, where relevant. A full description of CELAVI data requirements may be found in Hanes et al.,^37^ and additional details are available in Section 1 of the Supporting Information.

In the baseline analyses, only gross process costs were used; process revenues were not included. Revenue streams, particularly for nascent technologies and developing supply chains, are more uncertain relative to process costs and can be highly variable due to their dependence on secondary material market conditions.^45^ Moreover, revenue data were available for only a few of the many processes included in this study (see Supplementary Section 2 for a complete list of the revenue data and sources used in this study). Revenue for processes in the forward and reverse supply chains was therefore modeled as zero for the baseline results presented in Section 4. To explore the effects of this simplification, a revenue-focused sensitivity analysis was performed. The revenue of each process in the reverse supply chain without available revenue data was calculated based on an assumed 10% profit (that is, revenues were calculated as 110% of the gross process cost), and the resulting supply chain was simulated. All revenues in the reverse supply chain were then varied by ±75%. Including non-zero revenue and varying the magnitude of the non-zero revenue did not result in a different supply chain configuration and did not affect the cumulative amount of material sent to landfill. The results of this revenue sensitivity analysis are discussed further in Section 4.2, with additional details in the Supporting Information.

For the flat glass case study, historic and projected demand information was required for commercial single-, double-, and triple-paned building windows and for utility-scale PV modules. Recovery pathways for other flat glass applications were omitted from the study to keep its scope manageable. The commercial building sector stock model (ComStock) was used to generate window glass demand from commercial buildings for the years 2000–2018 inclusive.^46^ Window glass demand was then projected forward through 2050 by assuming periodic window replacement and a constant building stock year-over-year past 2018. Historic glass demand for utility-scale PV modules was obtained from the U.S. PV Database maintained by the U.S. Geological Service.^47^ Projected glass demand was obtained from NLR’s Regional Energy Deployment Energy Deployment System (ReEDS) model using the 2023 Mid-Case Standard Scenario assumptions.^48,49^ Both the historic and projected demand datasets were in units of installed megawatt (MW) PV capacity; this information was combined with data on the mass of glass required per installed MW, accounting for PV technology changes over time.^50^ Installation of non-utility-scale PV was not modeled in this study, as it is expected that collecting glass from distributed PV at EOL would be more challenging compared to utility PV. Demand for window and PV glass was aggregated to the U.S. state level, and demand centers were modeled at state centroids. This simplification was done to avoid modeling the location of every commercial building in the contiguous U.S. (CONUS), which would have increased the model computation time beyond practical limits without adding to the potential insights. Landfills, cement plants, PV recycling facilities, flat glass manufacturing facilities, and glass wool manufacturing facilities were modeled at real-world locations.^51–54^ Facilities producing glass cullet or supplementary cementitious material (SCM) were assumed to be co-located with glass wool manufacturing and cement production facilities, respectively. All facility locations were assumed to remain the same during the CELAVI simulation period (2025–2050). We assumed fixed lifetimes of 20 years for PV panels^50^ and 30 years for windows;^55^ early replacements due to breakage, repowering, and other activities were not modeled. Material compositions were taken from Ovaitt et al.^50^ and Parker et al.^46^ for PV and windows, respectively. Finally, Ecoinvent data^56^ were used for the life cycle analysis (Supplementary Table S2) with the functional unit of the LCA being the mass of flat glass used in U.S. utility-scale PV and commercial building window life cycles—from cradle-to-grave or cradle-to-cradle—during the 2025–2050 period. A mass-based functional unit is well suited for this analysis because the primary objective is to compare alternative fates of a fixed demand for flat glass across the full system, rather than to evaluate the functional performance of individual end products. While different pathways deliver different services, the mass basis allows for a consistent and tractable comparison of the environmental and economic consequences of routing the same quantity of material through competing circular and linear pathways. Pathways’ cost data were taken from Farel et al.^57^ and other sources (Supplementary Table S1).

For the IO models, the data collection process started with building a simplified glass MFA (Figure 3) using information from the U.S. Geological Survey (Silica Minerals Yearbooks^58^), the Environmental Protection Agency (EPA),^59,60^ the United Nations (UN) Comtrade database,^61^ and other publications.^25,30,31,62^ Next, Bureau of Economic Analysis (BEA) data from 2002 (when published IO tables were more detailed) were used to disaggregate the glass sector in the 2012 BEA tables used by PIOCA and EMPLOY.^63^ The “nonmetallic mineral products” (NAICS 327) sector was separated into five sectors in both EMPLOY and PIOCA: “flat glass”, (NAICS 327211), “other pressed and blown glass and glassware” (NAICS 327212), “glass containers” (NAICS 327213), “glass products from purchased glass” (NAICS 327215), and “other nonmetallic mineral products” (which comprises the original “nonmetallic mineral products” minus the glass sectors). In EMPLOY, commodities from the 213A0 sector (“other nonmetallic mineral mining and quarrying”) and 325180 sector (“other basic inorganic chemical manufacturing”) were also disaggregated into 5 products (silica, limestone, soda ash, other nonmetallic, and other basic inorganic chemicals), and “cullet” was added as a new commodity (see Supplementary Figure S3). It was also necessary to add a new sector to represent recycling activities. This new sector was derived from a techno-economic analysis on PV recycling (see Supplementary Information Section 2). Glass prices were also collected from the Comtrade database to convert monetary values into physical ones and were validated with the MFA (the difference in total glass flows was lower than 2%). Table 1 summarizes SCORPIO’s main input data and their sources.

**Table 1:** Key Data Aspects Used to Define SCORPIO’s Material Flow Recovery Pathways and Their Spatial and Temporal Characteristics

Data Aspect	Data Source	Temporal Scope	Spatial Scope
Historic window glass demand	ComStock^35^	2000–2018	U.S. state-level
Historic PV module demand	U.S. PV Database^36^	1986–2021	U.S. state-level
Projected PV module demand	NLR ReEDS model^37,38^	2025–2050	U.S. state-level
Glass demand centers (buildings, PV modules installed)	Rows 1–2	Rows 1–2	U.S. state centroid-level
Flat glass recovery in CELAVI	Output of CELAVI	2025–2050	CONUS
Glass MFA	USGS,^44^ , US EPA,^45^ , UN Comtrade^46^	2012–2022	U.S.
Glass IO tables	BEA	2019–2050	U.S. state-level

Scenarios for flat glass recovery were first modeled in CELAVI to understand changes within the glass supply chain. The supply chain scenario results were then fed into the IO models to determine the broader economic impacts. The geographical and temporal scopes for CELAVI were CONUS and the 2025–2050 period, respectively. The IO models’ geographic scope is the entire United States, including Alaska and Hawaii, and the temporal scope is 2019–2050.

In addition to the linear *Landfilling* pathway in which flat glass is sent to a landfill at the end-of-life (EOL), several additional material recovery pathways for flat glass were modeled (Figure 2):

*Closed-loop window refurbishment*: In this process, glass panes are recovered whole from building windows at EOL, cleaned, and reused in new frames.
*Closed-loop PV manufacturing*: In this process, glass is removed during PV module disassembly at EOL, ground into cullet, and reintroduced as a raw material into the PV manufacturing supply chain.
*SCM manufacturing*: In this open-loop (i.e., downcycling) process, glass waste from either building windows or PV modules is recovered (removal and/or disassembly), finely ground, and used by cement manufacturers as a supplementary cementitious material (SCM). PV modules require a preceding module disassembly step.
*Glass Wool Manufacturing*: In this open-loop process, glass waste from either building windows or PV modules is recovered (removal and/or disassembly), ground into cullet, and used as a raw material in glass wool manufacturing.

**Figure 2:**
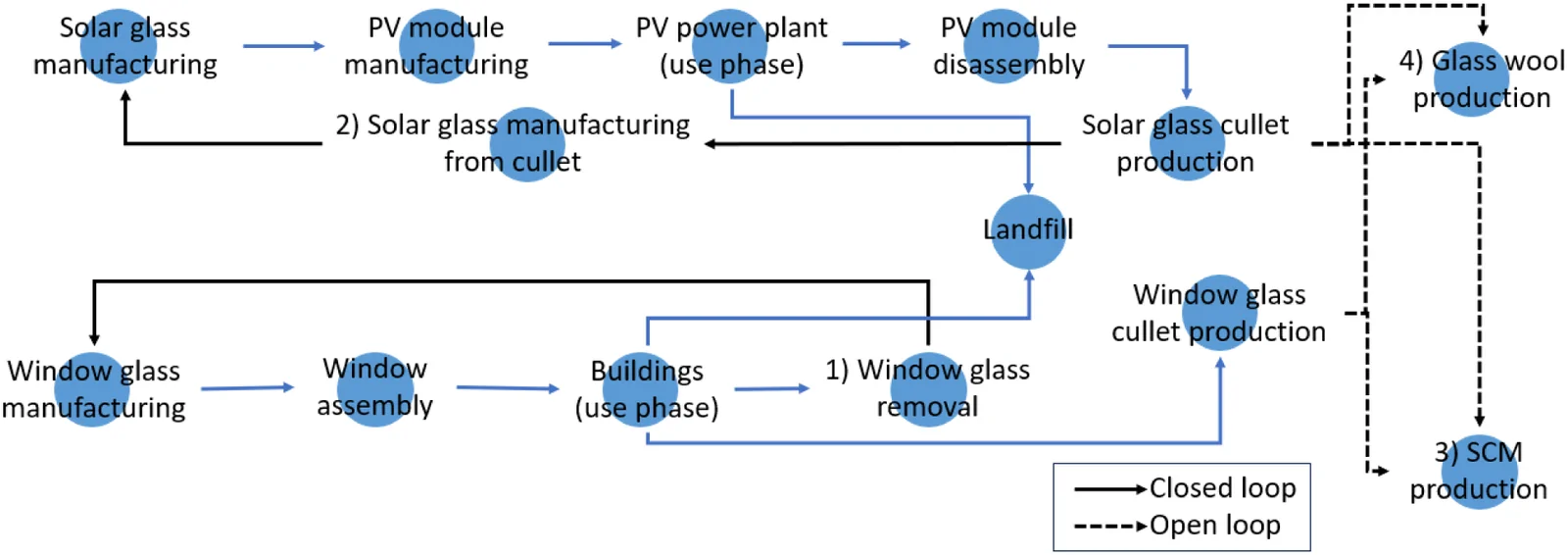
Simplified diagram of the supply chain superstructure modeled in SCORPIO. Some processing steps and other aspects of the supply chain were modeled but are not shown in this figure: material losses, natural resource extraction, PV module uninstallation, window uninstallation, transportation between steps, and material inputs other than glass. Numbers correspond to the four material recovery pathways described above.

In all material recovery pathways, system expansion with substitution assumptions (with substitution rates of 1:1 by mass) was used, in line with other studies.^64,65^ Supporting Information section 2 provides more details on the subprocesses and assumptions used for these material recovery pathways.

## 3 Results and Discussion

### 3.1 Glass Flows in the United States between 2012 and 2022

Figure 3 illustrates the complete material flow cycle of the U.S. glass manufacturing industry based on cumulative production data over the 2012–2022 period. The figure shows both the primary production supply chain and current recycling efforts of glass manufacturing. Around 180 Mt of glass were produced in the U.S. during the 2012–2022 period, with container glass dominating at about 85 Mt (48% of total production), followed by flat glass at 70 Mt (39%), and specialty/fiberglass products at 24 Mt (13%). Raw material inputs include silica (97 Mt), soda ash (17 Mt), and limestone (13 Mt). Pre- and post-consumer cullet account for just 30 Mt of the sectors’ material input (19%), demonstrating the industry’s reliance on virgin materials. Figure 2 also reveals that most glass ends up in landfills or low-value applications (e.g., landfill caps, construction aggregates). This is particularly true for flat glass (with a 5% recycling rate), indicating substantial opportunities to recover glass for higher-value secondary applications.

**Figure 3:**
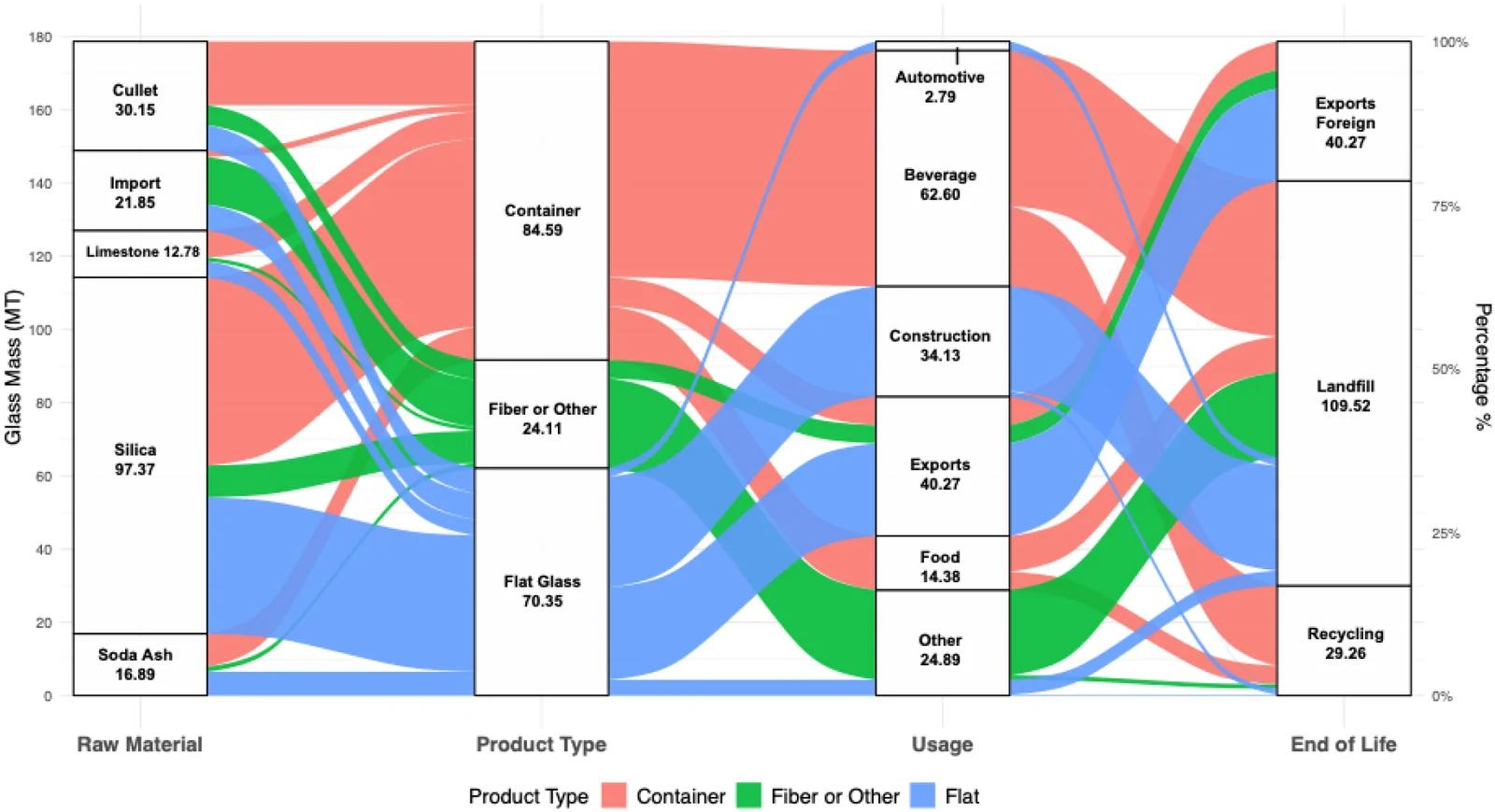
Sankey representing the total (i.e., cumulative) flows of glass from 2012–2022 in the U.S. economy, in metric tonnes. Yields from raw materials to glass products are already accounted for in the raw materials inputs (flat glass = 72%; container glass = 77%; other glass = 92%). Data table informing this Sankey is presented in Supplementary File 2.

To investigate those opportunities and answer the research questions defined in the methodology, we first conducted a sensitivity analysis to find break-even conditions where recovery of secondary flat glass approaches cost parity with *landfilling*. For each scenario, costs and revenues for each supply chain actor are considered separately—in order for a recovery pathway to receive market share, it must be profitable for all parties. Next, specific scenarios are analyzed in greater detail, looking both at changes in the U.S. glass supply chain and at impacts on the broader U.S. economy.

### 3.2 Reaching Break-Even with Landfilling: A Sensitivity Analysis

The baseline scenario process costs used within CELAVI reflect present-day conditions, and as a result, the *landfilling* EOL pathway is the lowest-cost option and the only one with market share in the baseline scenario. We assessed what level of process cost reductions would be necessary for the material recovery pathways described in Section 3.2 to become cost-competitive with the *landfilling* pathway by conducting a sensitivity analysis on material recovery pathway costs. The costs of processes involved in the glass circularity pathways (e.g., cost of PV and window disassembly, cost of glass beneficiation) were reduced to 50%, 10%, 1%, 0.1%, and 0% of their baseline values (Supplementary Table S1), and CELAVI simulations were run at each material recovery cost level. All other parameters within the flat glass supply chain were left unchanged, including transportation costs and virgin manufacturing costs.

The causes of these cost reductions were not explicitly represented. Cost reductions could derive from a combination of specific factors, including investments in recycling technologies (e.g., through the purchase of better sorting equipment, such as laser-induced breakdown spectroscopy^66^ or automated disassembly lines^67^), research and development (R&D) into material recovery processes, and policy mechanisms aimed at incentivizing recycling. Cost reductions could also be achieved in the long term through industrial learning-by-doing and potentially through economies of scale, as higher-capacity material recovery facilities come online.^68,69^ Chen and Jin,^68^ for instance, studied how optimized waste management systems could reduce energy costs in the pulp and paper sector. However, the very low cost reductions (e.g., 0.1%, 1%) included in this sensitivity analysis are not expected to be realized in the near future but enable the identification of cost-based tipping points at which the cost-competitive end-of-life pathway changes from landfilling to a circular pathway.

As described in Section 3, process revenues were not modeled in the baseline set of results and the sensitivity analysis on process costs. Results of an additional sensitivity analysis exploring how including and varying the value of process revenues affects supply chain outcomes are given in Supplementary Table S6. No change in supply chain outcomes (choice of pathway implemented, cumulative material landfilled) was observed when revenue was included or the revenue magnitude varied compared to the baseline scenario with no process revenues. Additional sensitivity analyses (Supplementary Table S6, Supplementary Figures S17 and S18) show that processing costs are the most sensitive parameters, besides product lifetimes, and that landfilling fees interact more with recycling costs than transportation costs. Figure 4 presents the results of 54 recycling cost scenarios for PV glass, in which both the cost of PV module disassembly and the cost of secondary material production (for closed-loop PV glass, SCM, and glass wool) were varied. In each scenario, the market shares (in terms of million tons of glass) were determined for each EOL pathway. The results highlight the cost conditions required to enable the various recycling pathways to compete with the *landfilling* pathway for EOL management of PV glass. As Figure 4 illustrates, *closed-loop PV manufacturing* requires the PV panel disassembly and manufacturing-from-cullet costs to both be lower than 10% of their baseline values. Downcycling to glass wool requires similar cost conditions but is less effective at keeping materials in circulation. About 0.5 Mt of flat glass is diverted from landfill when costs are at 1% of their baseline for *glass wool manufacturing*, compared to 1.0 Mt diverted from landfill by the *closed-loop PV manufacturing* pathway under equivalent cost conditions (PV panel disassembly and manufacturing-from-cullet costs at 1% of their baseline). Downcycling solar glass to SCM is the recovery pathway with the largest opportunity: 2.4 Mt diverted from landfills under the same cost conditions (module disassembly costs and SCM manufacturing costs at 1% of their baseline). This observation holds even when pathway costs for all material recovery pathways are reduced simultaneously (Supplementary Figure S7).

**Figure 4:**
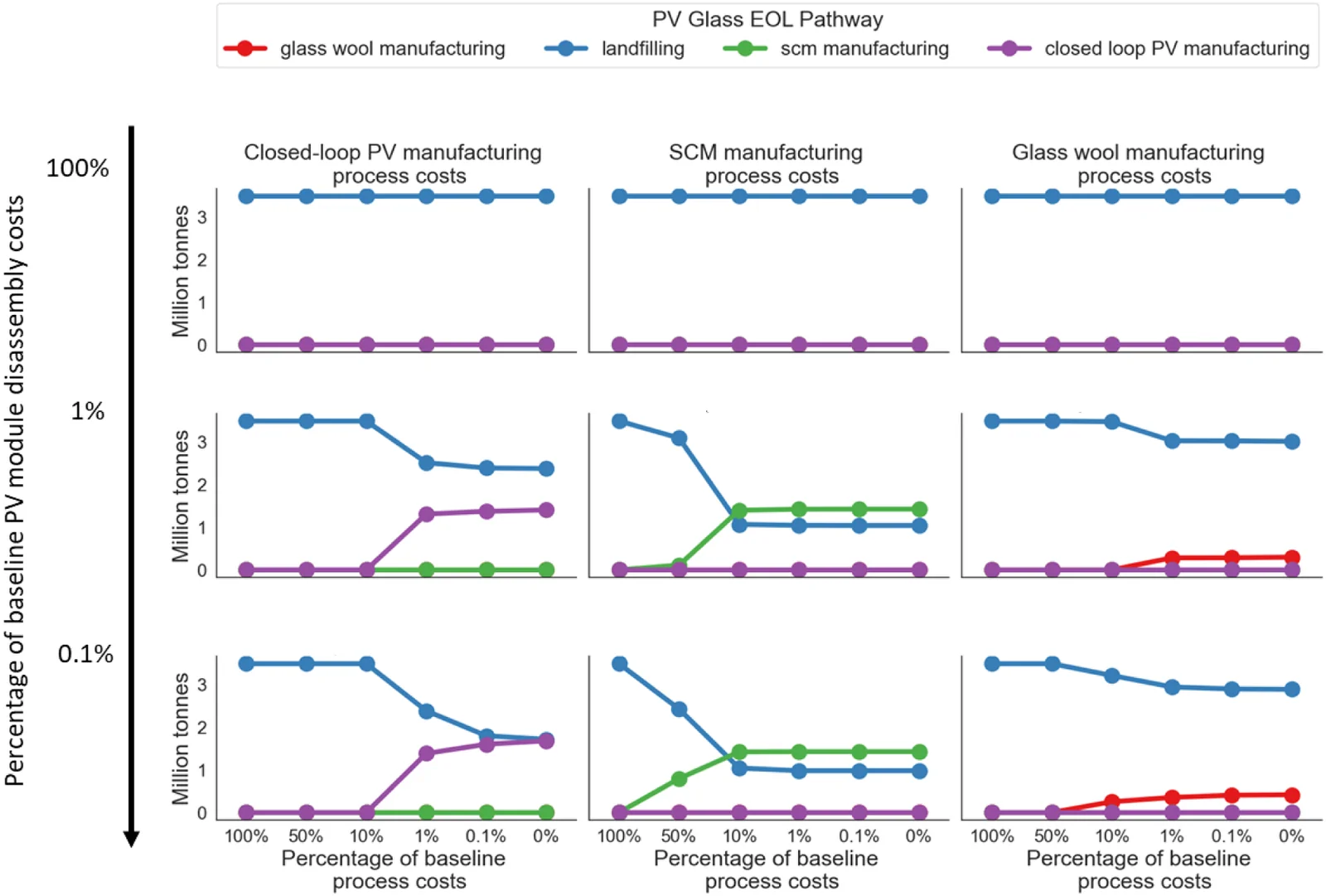
Sensitivity analysis for 54 scenarios (3 PV module disassembly cost levels times 3 pathway-specific cost parameters times 6 cost levels for those parameters) of PV glass EOL management in which material recovery cost parameters are varied across four EOL pathways. The figure displays the cumulative mass flows in million metric tonnes of PV glass in 2050 for different combinations of cost parameter values. PV module disassembly costs 1% or below their baseline enables open-loop and closed-loop recycling. In addition to reduced disassembly costs, *closed-loop PV manufacturing* costs, *SCM manufacturing* costs, and *glass wool manufacturing* costs need to be below 10%, 50%, and 50% of their baseline values, respectively, to gain appreciable market shares.

Similar sensitivity analyses were also performed for the recovery of commercial building window glass (Supplementary Figures S8 and S9). The cost of *closed-loop window refurbishment* is dominated by the window pane recovery step (i.e., disassembling windows prior to a buildings’ demolition), which must have its cost reduced to less than 1% of the baseline value to drive market uptake (Supplementary Figure S8). Reductions in the subsequent cost of window manufacturing do not have an appreciable impact. Meanwhile, the scenarios for open-loop recovery of window glass show similar results to the equivalent scenarios for PV glass (Supplementary Figure S9). Downcycling window glass to SCM is once again the recovery pathway with the largest waste diversion opportunity, while *glass wool manufacturing* has a lower market cap.

For both PV and window glass, the sensitivity analyses also demonstrate that every material recovery pathway exhibits diminishing returns on how much material is diverted from landfills as recovery pathway costs are reduced; in other words, market uptake plateaus. These diminishing returns are due to transportation costs that are constant across all sensitivities. Since landfills are more numerous and more widely distributed than cement factories, glass wool manufacturing plants, and PV recycling facilities, transportation costs from the PV or window decommissioning site to the closest landfill will often be lower than those for the other pathways. For the same reason, because there are more cement factories than glass wool manufacturing and PV recycling plants, the SCM pathway plateaus at a higher market share than that of the glass wool and closed-loop pathways. While transportation costs were not varied in the cost sensitivity analyses, we would expect that other factors, such as the construction of additional processing facilities and the development of secondary material revenue streams, could impact transportation costs or diminish their importance.

In the next sections, the potential benefits of increased glass recovery will be further explored by comparing the economic and environmental impacts of key reverse supply chain steps in three selected scenarios for PV glass recovery:

The *Baseline* scenario, in which all supply chain costs are left at their baseline values;
The *PV-PV Closed-Loop* scenario (module disassembly costs = 1% baseline; PV glass manufacturing from cullet costs = 1% baseline) and
The *PV-SCM Open-Loop* scenario (module disassembly costs = 1% baseline; SCM manufacturing costs = 10% baseline).

In the two material recovery scenarios, the highest parameter values enabling landfill diversion in Figure 4 were chosen (i.e., the scenarios are the closest to break-even conditions).

### 3.3 Supply Chain Impacts of PV Glass Recycling

Figure 5 shows the evolution of costs and revenues as PV panels are being decommissioned and enter reverse-supply-chain pathways in the *baseline, PV-PV closed-loop*, and *PV-SCM open-loop* scenarios. While those results should be taken cautiously given their dependence on cost and revenue assumptions (which can be highly variable depending on market conditions), they offer insights into the possible economics of the material recovery pathways. In the *Baseline* scenario (Figure 5a), all PV panels are sent to landfill at EOL, costing about $100 million in landfill fees and generating no revenues. In contrast, in the *PV-PV Closed-Loop* scenario (Figure 5b), manufacturing flat glass from cullet generates a significant revenue stream from the sale of the glass to PV manufacturers (about $1,300 million over the simulation period) with a total cost of approximately $900 million. This scenario also avoids about $30 million in landfill fees.

**Figure 5:**
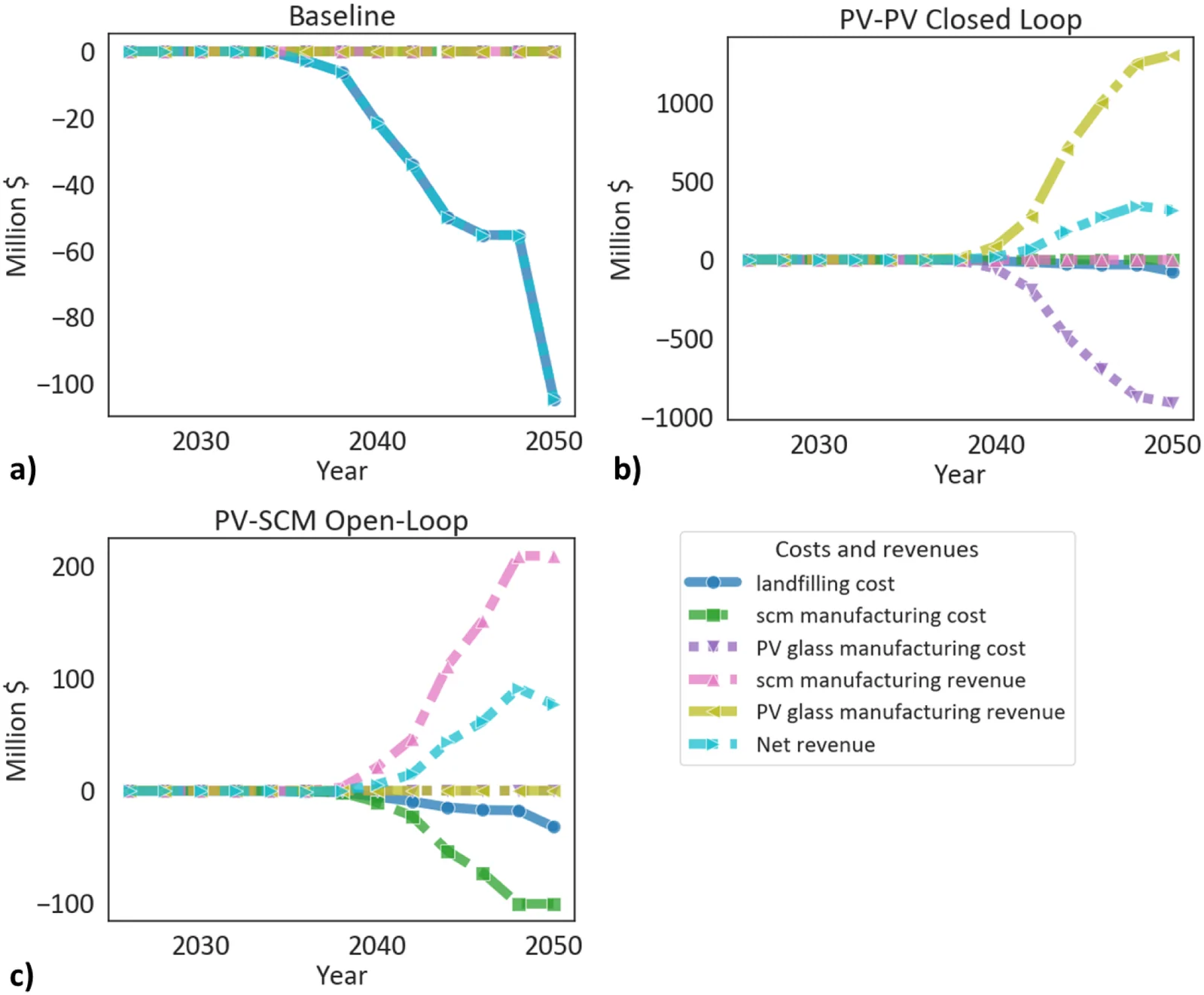
Costs, revenues and net revenue of the PV reverse supply chain steps in the *Baseline* (a), *PV-PV Closed-Loop* recycling (b), and *PV-SCM Open-Loop* scenarios.

In the *PV-SCM Open-Loop* scenario (Figure 5c), the steps required to transform the waste glass into a pozzolan-like cement replacement material cost $100 million, while selling the SCM to a concrete manufacturer generates $200 million in revenues. In that scenario, $70 million in landfill fees is avoided. Hence, although the *PV-PV Closed-Loop* and *PV-SCM Open-Loop* recycling scenarios both require module disassembly costs to be significantly lower than what they are currently, they both provide economic benefits for downstream flat glass supply chain actors. Supplementary Table S3 and Supplementary Figures S10 and S11 present a complete picture of the costs and revenues for each of the supply chain actors for the different scenarios.

In addition to causing economic impacts, material recovery pathways can both lead to and avoid environmental impacts due to an increase in recycling activities and a decrease in virgin material extraction and processing, respectively. Thus, an LCA conducted with CELAVI was used to calculate a range of environmental impacts associated with specific steps in the PV glass supply chain. As an example, Supplementary Figure S12 shows the distribution of particulate matter emissions from U.S. landfill sites in the *Baseline* and *PV-PV Closed-Loop* recycling scenarios. Unsurprisingly, impacts linked to *landfilling* are reduced in the material recovery scenario compared to the *Baseline*—especially in states that will have high volumes of decommissioned PV panels in the coming years and that have nearby PV recycling infrastructure (e.g., California). However, when considering the entire PV supply chain, environmental impacts are mostly similar between the *Baseline* and *PV-PV Closed-Loop* recycling scenarios (Supplementary Table S4). This result is explained by relatively high additional transportation and energy-use impacts from recycling balancing out the avoided energy and virgin material impacts of using cullet in flat glass manufacturing.

When comparing with the *PV-SCM Open-Loop* scenario, Supplementary Figure S13 shows that more glass waste is diverted from landfills in the *PV-SCM Open-Loop* than the *PV-PV Closed-Loop* recycling scenario (2.4 Mt vs. 1 Mt). As a result, greater reductions can be seen in landfill particulate-matter emissions. The figure also indicates that although landfill impacts are reduced, the *PV-SCM Open-Loop* scenario does have greater particulate-matter emissions from cement manufacturing plants, where the pulverization of glass into SCM is likely to occur. However, those additional impacts are somewhat balanced by a reduction in impacts due to the lower demand for Portland cement manufacturing (Figure 6 and Supplementary Table S4). Overall, the *PV-SCM Open-Loop* recycling scenario avoids 307 Mt of PM 2.5 eq. Moreover, the *PV-SCM Open-Loop* scenario seems to cause less environmental and human health impacts than the *PV-PV Closed-Loop* recycling scenario, with more impact categories that are lower or equal to the *Baseline* in Supplementary Table S4.

**Figure 6:**
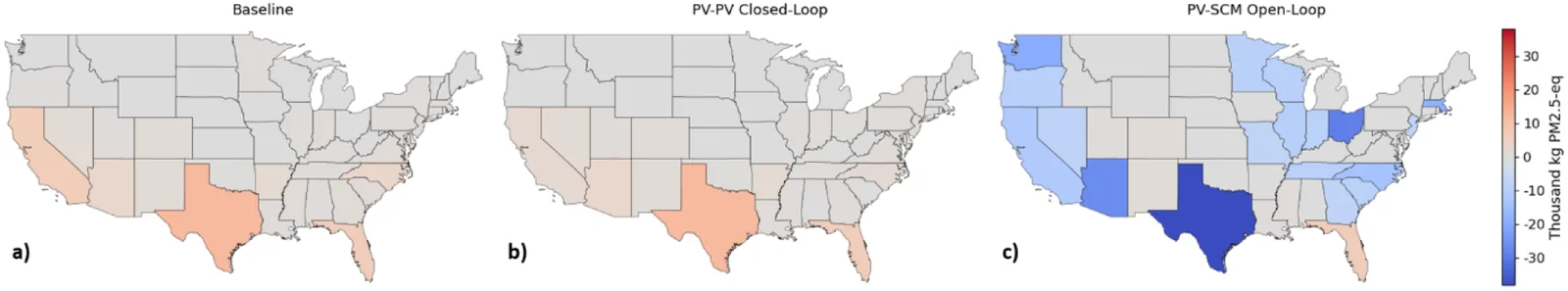
Distribution of particulate matter emissions (respiratory effects category in TRACI) in the *Baseline* (a), *PV-PV Closed-Loop* recycling (b), and *PV-SCM Open-Loop* recycling (including avoided impacts from cement manufacturing) (c) scenarios. Values are cumulative over the whole simulation period (2025–2050) and only the impacts from the reverse supply chain processes are shown (see Supplementary 4 for the whole life-cycle impact values).

### 3.4 Economy-Wide Impacts of PV Glass Recycling

Next, SCORPIO’s economy-wide assessment capabilities were leveraged to assess the economic and workforce changes throughout the U.S. economy in the *PV-PV Closed-Loop* scenario. For this analysis, an additional scenario assuming ideal PV recycling conditions (*100% PV-PV Recycling*) was used for comparison (Supplementary Figure S14).

Figure 7 Cumulative economy-wide GDP and workforce impacts of these two scenarios from 2025–2050. In the *PV-PV Closed-Loop* scenario, SCORPIO estimates that over the entire U.S. economy, about $497 million in net additional gross domestic product (GDP) per year would be generated and around 3,800 net jobs would be created by the end of the 2025–2050 period (Figure 7a and 7b). About one-third of those new jobs would be indirect, mainly in the transportation and chemical sectors, which support PV recycling activities. Unsurprisingly, the PV recycling sector itself sees the largest positive changes in GDP and employment, while the waste management sector, which includes landfills, experiences the largest negative changes in those same indicators. In this scenario, the supply of cullet from solar glass recycling reaches a maximum of 13% of U.S. flat glass production in 2050 (Supplementary Figure S14). One reason is that PV panels have long lifetimes and stocks are only just starting to grow.^70^ Hence, there is a high discrepancy between the amount of flat glass within PV panels that are still in their use phase and the amount of flat glass waste generated from decommissioned PV panels during the 2025–2050 period (Supplementary Figure S15).

**Figure 7:**
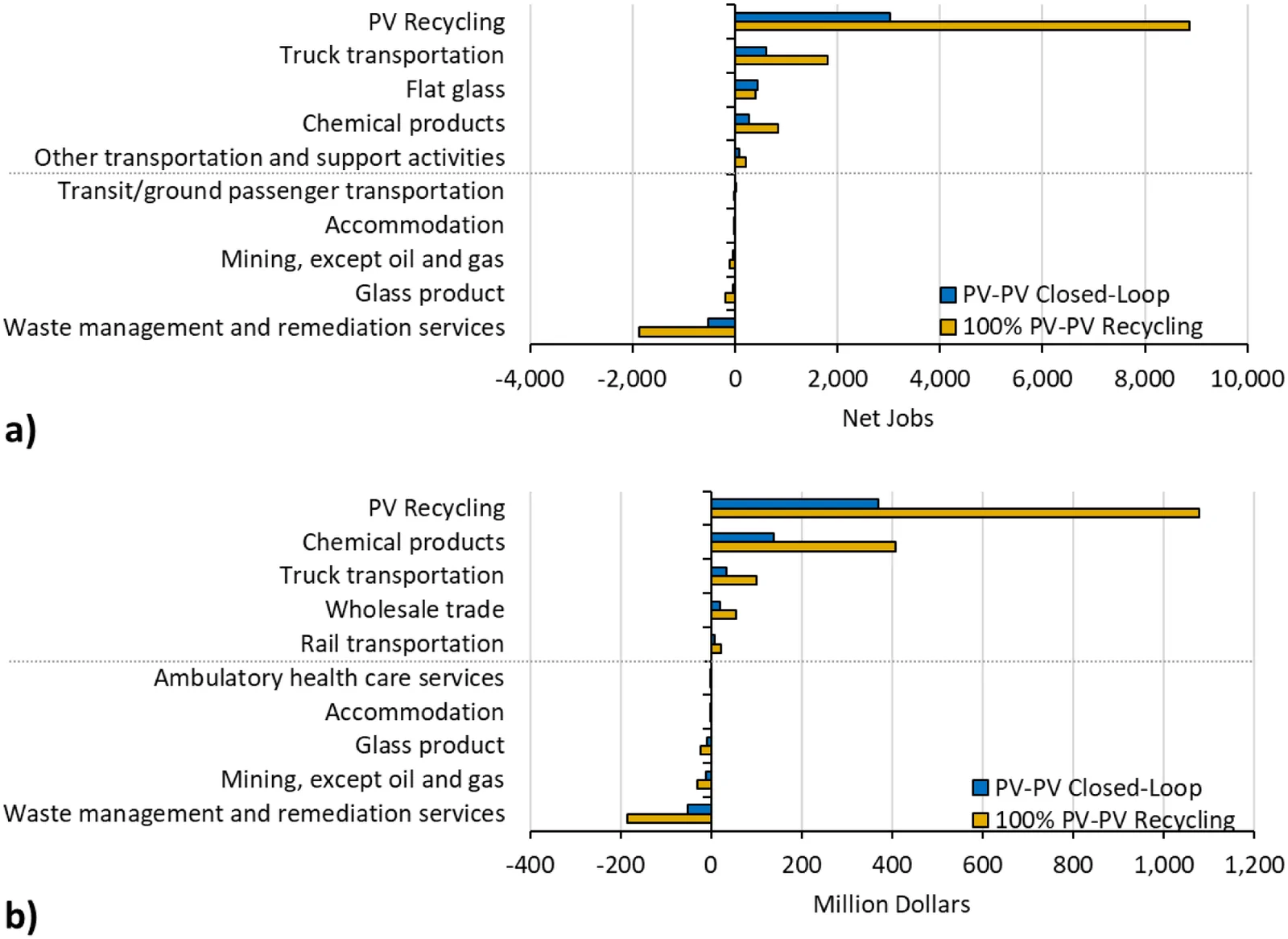
Workforce (a) and economic (b) results from the economy-wide assessment of CELAVI’s *PV-PV Closed-Loop* and *100% PV-PV Recycling* scenarios.

To provide more perspective, the impacts of ideal PV recycling conditions are explored in the 100% PV-PV recycling scenario. In that scenario, about $1.4 billion of net additional gross domestic product (GDP) per year would be generated, and around 10,000 net jobs would be created by the end of the 2025–2050 period (Figure 7a and 7-b).

### 3.5 Structure of Material Flows within the Economy and Effect of Increased Material Recovery

Besides their economic, environmental, and workforce impacts, material recovery pathways may also alter the structure of material flows within the economy. SCORPIO’s physical IO model was used to capture those structural changes in CELAVI’s *PV-PV Closed-Loop* and the ideal *100% PV-PV Recycling* scenarios. Figure 8 illustrates the positive and negative changes relative to the *Baseline* scenario in material (Figure 8a) and monetary flows (Figure 8b) between economic sectors in the *PV-PV Closed-Loop* scenario. Mining commodities are not demanded as much by the flat glass sector in the *PV-PV Closed-Loop* scenario as in the *Baseline* scenario, as seen by the negative value going from the mining sector (y-axis) into the flat glass sector (x-axis) in Figure 8a. Moreover, Figure 8 shows that while the ∼1.0 Mt of glass cullet generated in the closed-loop recycling scenario (Figure 4) represents the largest change in material flow—as shown by the positive value going from the flat glass cullet sector (y-axis) into the flat glass sector (x-axis) in Figure 8a—it is only part of broader changes within the U.S. economy. Mass flow changes between the manufacturing sector and other sectors are also significant, highlighting the intricate relationship between industrial sectors, where many materials are processed and used in multiple sectors. Overall, mass flows from manufacturing and other industrial sectors to the waste management sector are reduced, confirming that material recovery reduces the direct and indirect waste associated with the flat glass supply chain. Supplementary Figure S16 illustrates similar results for the 100% PV-PV Recycling scenario, with a greater magnitude of changes.

**Figure 8:**
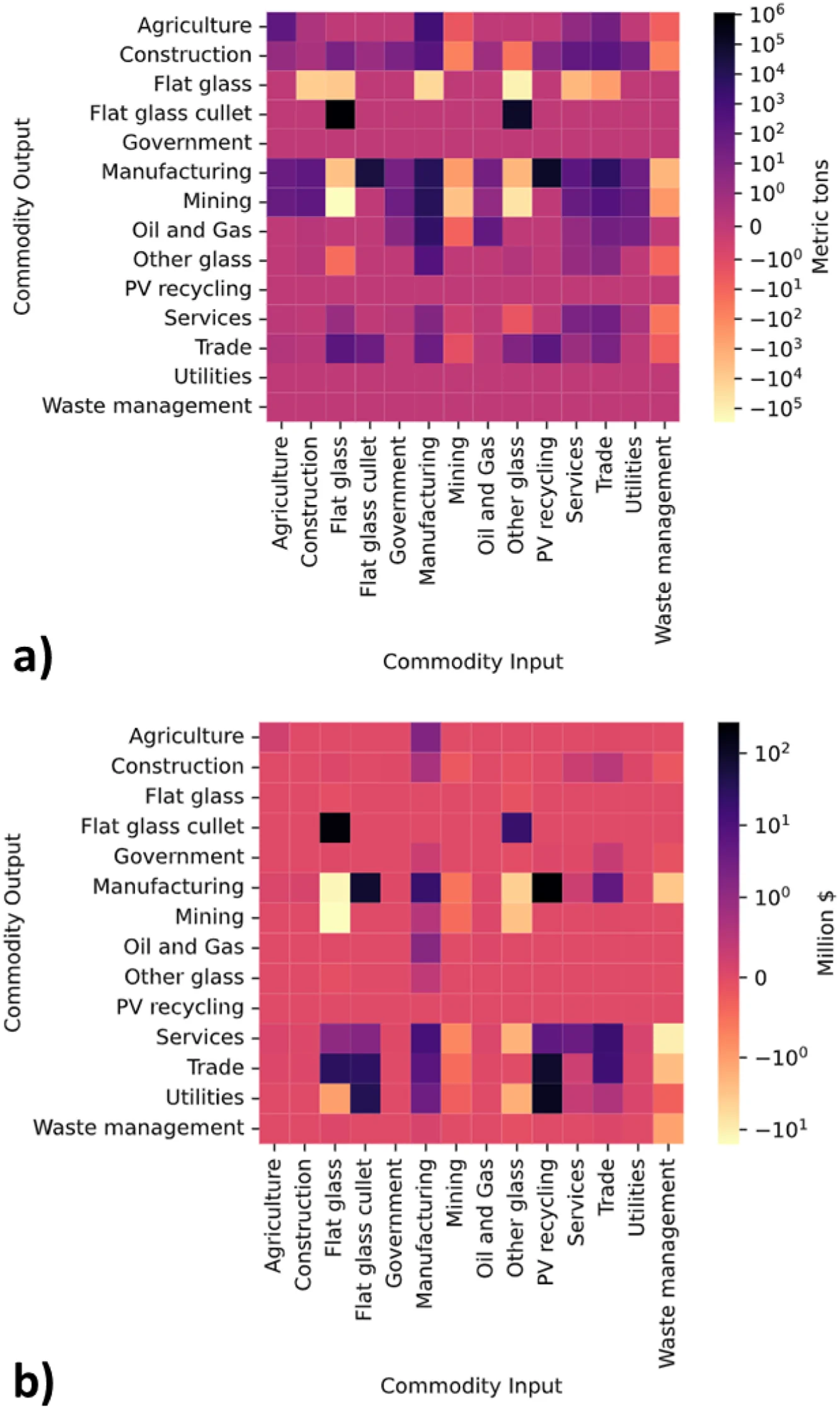
Changes in the U.S. economy between CELAVI’s *PV-PV Closed-Loop* and *Baseline* scenarios as reported by SCORPIO’s a) physical IO tables, b) monetary IO tables. Note that economic sectors have been aggregated for clarity in this figure (Supplementary Table S5).

Comparing Figure 8a and Figure 8b also highlights that while general trends in intersectoral flows remain the same when using monetary accounting of the economy, several structural changes are underestimated and others are overestimated relative to the material flows (due to differences in prices per mass unit). For instance, the flow of flat glass cullet into the flat glass sector is still one of the largest changes brought by the material recovery scenario compared to the *Baseline*, with $214 million going from the flat glass cullet sector (y-axis) into the flat glass sector (x-axis) in Figure 8-b. However, other significant changes in material flow are not apparent from the monetary flows, especially in the manufacturing sector. For example, the increase in mining sector flows into the manufacturing sector has a relatively low magnitude in monetary flows. The reduction in output from the mining sector into the flat glass sector is also one of the main negative changes in monetary accounting, with $16 million going from the mining sector (y-axis) into the flat glass sector (x-axis) in Figure 8b. However, the reductions in flat glass flows in other sectors are of relatively lower magnitude than those in the physical accounting. Finally, the changes in high-value commodities going from the services, trades, and utilities sectors to the other sectors of the economy are of greater relative magnitude in monetary accounting, in both directions (positive and negative).

## 4 Discussion

The consistent appearance of plateaus in Figure 4 (and Supplementary Figures S8 and S9) indicates that virtually all material recovery pathways reach a point at which additional cost reductions would have little to no effect on material flows. These results can therefore be used to inform on the minimum level of cost reductions—achieved, for instance, through R&D investments—that might be necessary to see material recovery pathways displace *landfilling,* as well as the cost-reduction level past, which no additional material recovery is achieved. In other words, SCORPIO’s supply chain analysis component can help inform decision makers on what level of technological investments are necessary to break even with the *landfilling* pathway, but also on when material recovery pathways become unresponsive to additional cost reductions. Taken together, this information enables estimating cost reduction targets for each of the material recovery pathways.

For the SCM open-loop recycling pathway, those conditions occur at a module disassembly cost equal to 1% of its baseline (∼$14/Mt) and an SCM manufacturing cost equal to 10% of its baseline (∼$7/Mt). This corresponds to a cost reduction target of $3.6 billion over the 2025–2050 period—for 2.4 Mt of glass waste diverted from landfills, or $1,500/Mt. For PV-to-PV glass recycling, those conditions occur at a module disassembly cost and a solar glass manufacturing-from-cullet cost equal to 1% of their baselines. This corresponds to a cost reduction target of $2 billion over the 2025–2050 period—for 1 Mt of glass waste diverted from landfills, or $2,000/Mt.

Downcycling flat glass to SCM is the most feasible material recovery pathway among those examined in this study, due to relatively low-cost process technologies, an existing market for SCM, and minimal contamination or material quality concerns. In other words, it is the pathway most likely to achieve a significant level of waste diversion with the minimum level of investment. PV glass closed-loop recycling is also an interesting pathway, as it has the potential for the highest revenues within the flat glass reverse supply chain. However, deploying this pathway within the U.S. would require the development of glass recycling technologies that can process both *rolled glass*—which is much of the flat glass used in c-Si PV panels today—and *float glass*, which is the type of flat glass currently produced in the U.S.^71^ Rolled glass manufacturing lines are common in countries that manufacture PV panels for export to the U.S., but are not present domestically, limiting PV glass recycling. Research into rolled flat glass recycling technologies is ongoing^72^ and while closed-loop recycling of flat glass from PV panels is unlikely to be widely deployable in the short term, establishing this material recovery pathway within the U.S. could lead to fully domestic and circular PV manufacturing supply chains.

Cost reductions of around $4 billion over the 2025–2050 period in the flat glass waste sorting and recycling processes could enable breaking even with the linear status quo and generate significant revenues for glass beneficiation and SCM manufacturing companies. However, as the plateaus in Figure 4 demonstrate, logistical costs also need to be addressed to maximize material recovery. This barrier to circularity and material recovery is well known and has been identified in numerous studies on very different technologies,^73–75^ highlighting its ubiquitous nature. While SCORPIO may be used to identify material recovery investment opportunities, the estimates provided in this study are likely inflated, since only material recovery costs (and not revenues) were used in the baseline supply chain simulations. As discussed in Section 3, this modeling choice was motivated due to the high uncertainty in the different material recovery pathway revenues and the lack of process revenue data, particularly for processes in the reverse supply chain. The impact of this limitation has been assessed with separate sensitivity analyses on process costs and process revenues (see Supplementary Table S3 for raw cost and revenue data, Supplementary Figures S9 and S10 for results of the sensitivity analysis on costs, and Supplementary Table S6 for results of the sensitivity analysis on revenues). Therefore, our estimates of the cost break-even points for secondary pathways represent lower limits on processing costs; the same break-even points could be reached through a combination of lower processing costs and stronger secondary material markets that increase revenue. Further steps of this research could be to collect better revenue data and modify the shortest-path algorithm to refine the analysis presented therein. A preliminary sensitivity analysis indicates that similarly to Figure 4 where significant cost reductions are needed to increase the flat glass circularity rate, high revenue streams would be necessary to obtain the same effect (Supplementary Table S6).

This study also showed that although both open-loop and closed-loop material recovery pathways have the potential for positive economic impacts at the supply-chain and economy levels, their effects on environmental impacts are more mixed. Most material recovery pathways resulted in small environmental impact reductions. However, among the material recovery pathways considered, SCM recycling offered the greatest potential for environmental impact reductions. This insight is in line with other studies that have found that open-loop recycling is sometimes preferable to closed-loop recycling,^8^ including in the glass sector.^76^ However, those conclusions remain sensitive to allocation assumptions.^64,65^

The economy-wide assessment results show that, from a system perspective, at least part of an initial investment in sorting and recycling technologies would be recouped by growth in GDP while also benefiting local communities through new job prospects. Overall, the negative changes that occur in some of the economic sectors (e.g., mining and waste management) are relatively smaller than the positive gains from increased material recovery in the flat glass supply chain. However, as in the supply chain analysis, logistics seems crucial as the physical flows between many sectors of the U.S. economy are radically altered by increased material recovery (Figure 8). The case study results also confirm that a physical accounting of the economy is necessary to appropriately assess the structural changes caused by material recovery, as suggested by Singh et al.^77^ and others.^78,79^

This study made several assumptions that require further investigations. First, all facility geographical locations of the forward and reverse supply chains are assumed to remain the same during the simulation period. Figure 4, for instance, might underestimate the potential for material recovery since, as recycling volumes grow, new recycling facilities could be built to meet demand, reducing transportation costs and raise the plateaus observed in the figure. Moreover, glass beneficiation facilities, which produce glass cullet and pozzolan-like SCM, are assumed to occur on the site of the end-user (e.g., pozzolan manufacturing is assumed to occur near cement facilities). Only utility-scale solar installations were considered, which means that a significant share of PV panel waste was not taken into account. This is a reasonable simplification since utility-scale PV currently represents 2/3rd of all PV installations and is expected to dwarf commercial and residential installations in the future.^49^ Besides flat glass used in commercial and residential PVs, this study also omitted flat glass used in the automotive sector and residential buildings. Moreover, while we describe possible mechanisms, the cost reduction assumptions enabling material recovery were not grounded in published technology roadmaps or previous analysis, which would have informed on what reductions are plausible within a specific time horizon. Given those limitations, the results presented in this study should not be generalized to the entire flat glass sector without further validation.

Although Supplementary Figure S17 shows that processing costs are the most influential parameters after product lifetimes, those parameters were varied one-at-a-time. Supplementary Figure S18 provides a first glance at some parameter interactions within the model, but additional uncertainty analyses, such as Monte Carlo simulations, would be necessary to fully capture interaction effects between parameters, providing further insights into the potential for flat glass material recovery in the U.S. Supplementary Figures S17 and S18 nonetheless confirm that for PV glass, the SCM open-loop recycling pathway may be more easily implemented in the U.S. if its supply chain costs are reduced than the closed-loop pathway.

This study assumes fixed direct emission coefficients for industrial processes over the 2019–2050 study horizon, which does not account for potential changes in the emission intensities of the industrial processes themselves. It should be noted, however, that emission intensities within the sector are not entirely static. The electricity grid mix evolves over time, as informed by the underlying ReEDS scenarios used to define the electricity sector, and since electricity represents a significant energy input for many of the processes modeled, a meaningful share of total emission intensity does change endogenously across the study time horizon. This assumption is acknowledged as a limitation of the current analysis. Moreover, while SCORPIO models uncertainty in LCA input parameters, it does not account for uncertainty in emission factors for LCA processes (e.g., using ecoinvent’s pedigree scores). Further work could add such emission uncertainty factors in the SCORPIO models.

This study demonstrates how SCORPIO, a multi-scale, multi-indicator material recovery framework, could provide a comprehensive view of the benefits and trade-offs of increased material recovery and beneficiation within supply chains. To the best of our knowledge, this is the first time a detailed and dynamic supply chain model has been combined with both monetary and physical IO tables, enabling the generation of new types of insights. This novel framework was applied to a case study on the flat glass sector to understand what level of cost reductions would be necessary to divert flat glass waste from landfills and to explore how economic and environmental impacts might change under different material recovery strategies—both at the supply chain and economy-wide levels. While open-loop recycling seems to be the most attractive solution for flat glass in the U.S. context, further research could alleviate some of the limitations of the present study.

## Supplementary Material





## Notes

Additional methodological elements and results (PDF)
*Sankey Diagram & PV Recycling Facility*: Excel document providing additional data used in the study (XLXS)
