# Supplementary material for: Multi-Scale Multi-Indicator Assessment of Material Recovery Pathways: The Case of the United States Flat Glass Sector
Source: Environ Sci Technol. 2026 Oct 23;60(44):31517–32. doi: 10.1021/acs.est.6c01727 (PMC13757939; doi:10.1021/acs.est.6c01727)
Supplement: Supplementary file 1 [file es6c01727_si_001.pdf]

Supplementary Information to:

Multi-scale multi-indicator assessment of material recovery pathways: the case of the United States flat glass sector

Julien Walzberg†1, Nivedita Biyani*1, Shubhankar Upasani1, Mansi Sharma1, Liz Wachs1, Rebecca J. Hanes1, Tapajyoti Ghosh1, Andre Avelino1, Annika Eberle1, Alberta Carpenter1*

**J.W. and N.B. contributed equally to this paper.*

†Corresponding author: julien.walzberg@nlr.gov

1National Laboratory of the Rockies, 15013 Denver West Parkway, Golden, CO, USA 80401

1. **Additional methodological elements** 2
2. **Additional scenario details** 13
3. **Additional Figures and Tables** 18
4. **References** 34

1. Additional methodological elements

SCORPIO overview:

The Supply Chain Operations Research through Physical Input Output (SCORPIO) framework combines three existing models – the Circular Economy Life Cycle Assessment and Visualization (CELAVI) model, the Physical Input-Output for Circularity Analysis (PIOCA) model, and the Environmentally extended Multi-regional Projection of Lifecycle and Occupational energy futures (EMPLOY) model. Supplementary Figure 1 provides an overview of how those models are combined.

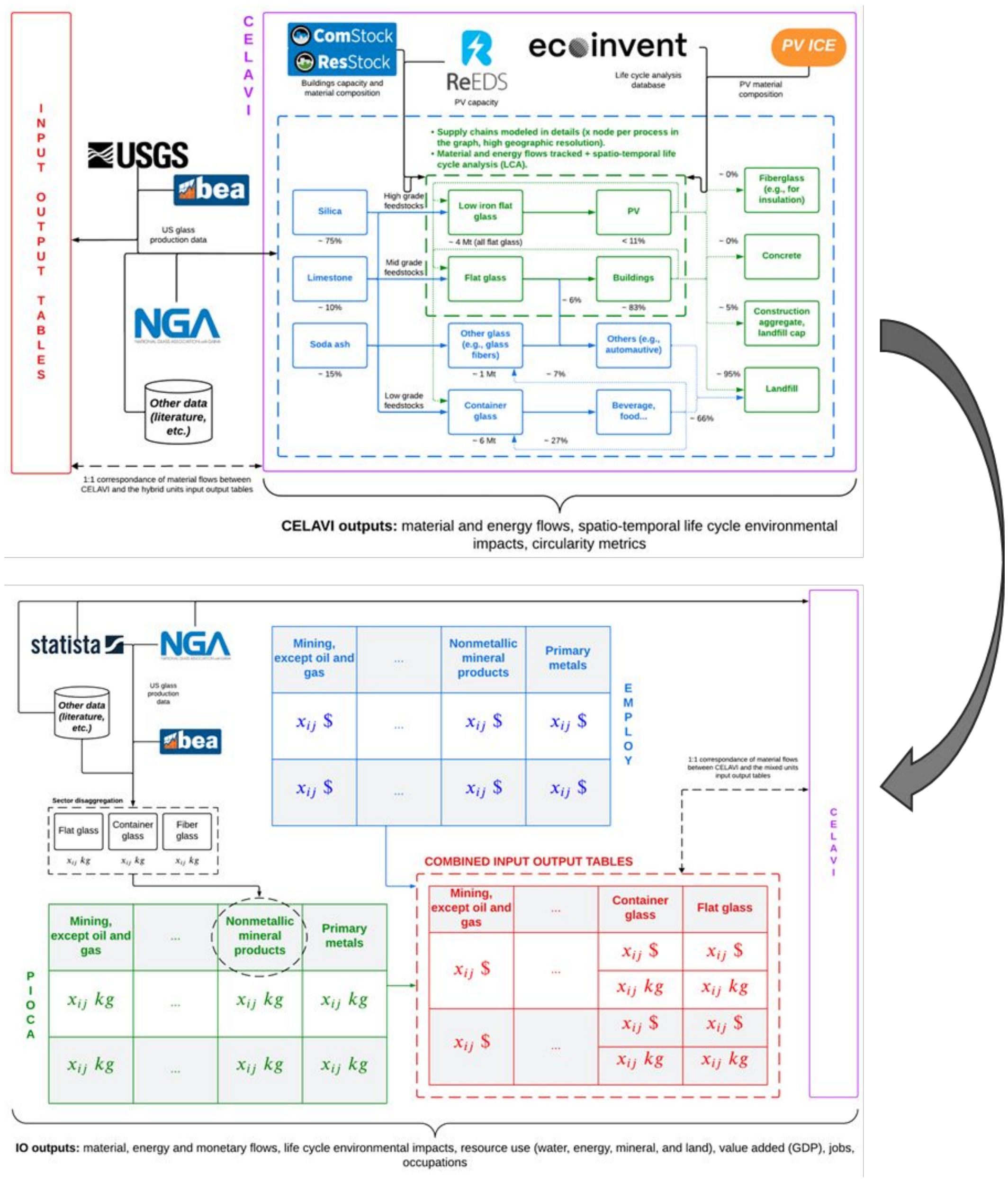

Supplementary Figure 1: Detailed representation of the SCORPIO framework showing input data sources.

CELAVI Model Description:

The CELAVI framework simulates supply chains as they transition towards circularity or remain linear, depending on the relative processing costs of linear and circular pathways, and computes associated environmental impacts and supply chain costs. It is data-driven and, thus, highly flexible. Any supply chain may be modeled if the technologies, materials, process technologies, and relevant facility locations are known. Moreover, Monte Carlo analysis can be used to evaluate uncertainty [1], although this was not done in the current work. CELAVI combines discrete event simulation (DES) for technology-component-level material flows; a life cycle assessment (LCA) module that uses a dynamic life cycle inventory derived from the ecoinvent dataset; and a network-based, spatially explicit model of the system's supply chain superstructure including all facilities, on-road transportation, and process cost models. CELAVI's structure is summarized in Supplementary Figure 2. CELAVI can include all life cycle stages of technologies and account for different end-of-life (EOL) options. For instance, a wind blade case study modeled two CE strategies, cement co-processing and mechanical recycling, along with landfilling [2]. The DES module within CELAVI calculates pathway costs at every time step, and routes EOL technology components along the least-cost pathway, creating a supply chain structure that changes over time with technology demands and process costs and varies spatially due to facility locations and transportation costs. The LCA module of CELAVI calculates dynamic, spatially resolved environmental impacts of the supply chain and its material flows. Thus, environmental impacts can be quantified at the facility level and for the entire supply chain. A range of metrics can be computed with CELAVI: several circularity metrics, life cycle environmental impacts, and the costs associated with different EOL pathways. This capability enables the framework to support multi-criteria decision making around material recovery pathway development. The model and the datasets used in this study are available on GitHub:

https://github.com/NatLabRockies/SCORPIO.

While CELAVI's facility-level, least-cost method for selecting EOL pathways offers more granularity than a system-level optimization, it does not currently consider heterogeneity in decision-making nor the possibility of conflicting objectives in different supply chain actors (e.g., between manufacturers and recyclers). For instance, non-cost-related challenges with material recovery pathways such as risk aversion are not explicitly captured. CELAVI is also not currently intended to model multiple, cross-sector supply chains, and cannot be used to quantify impacts in sectors outside the supply chain modeled or economic impacts such as net job creation.

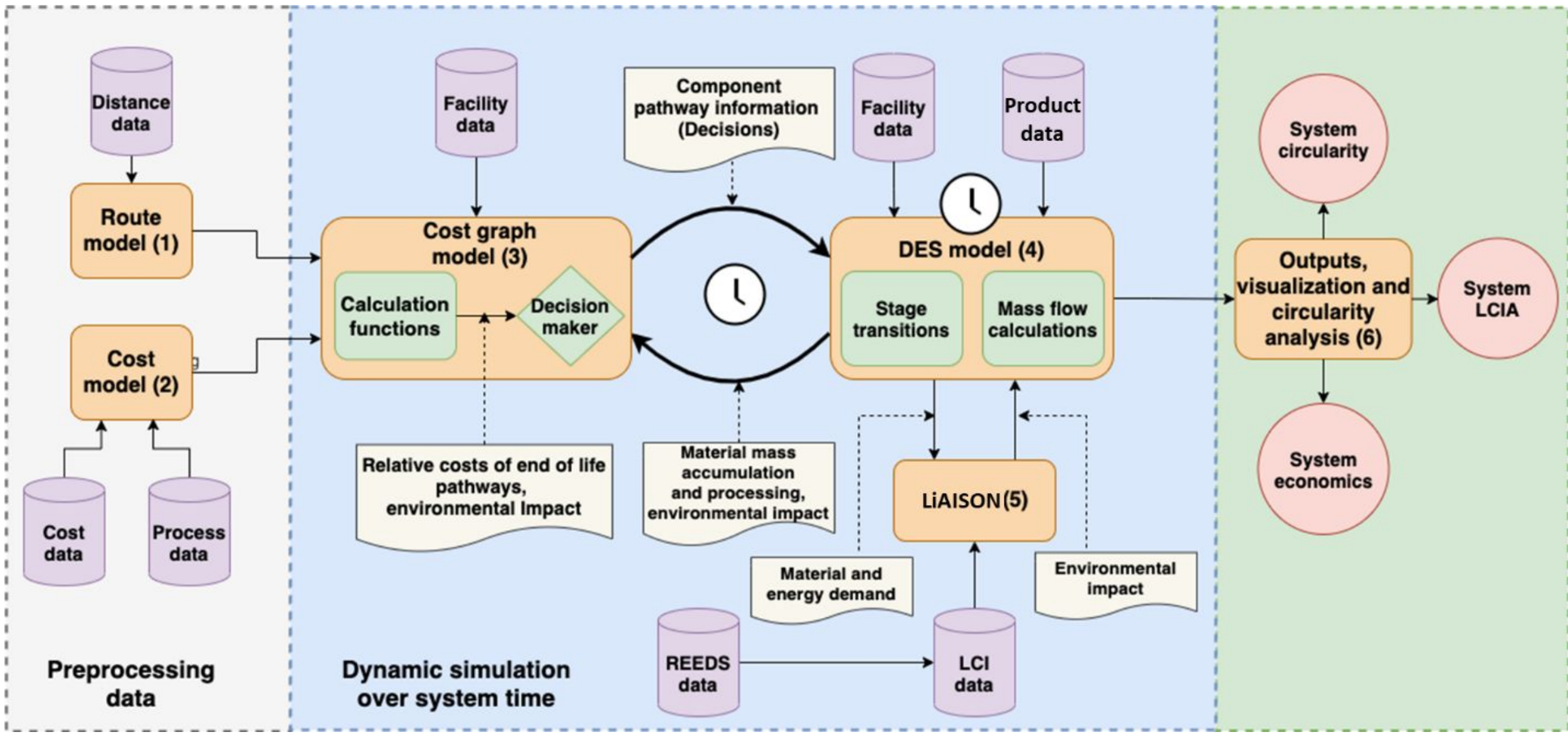

Supplementary Figure 2: Overview of the CELAVI model. Adapted from Hanes, Ghosh [2].

EMPLOY Model Description:

The Environmentally-extended Multi-regional Projection of Lifecycle and Occupational energY futures (EMPLOY) model is a combined environmentally-extended input-output (EEIO) and life cycle assessment (LCA) tool. It was designed to quantify the net impacts of a portfolio of technologies consistently across several economic, workforce, emissions and resource use metrics. EMPLOY performs "what-if" prospective scenarios (up to 2050) to estimate the net effects of different industrial deployment strategies and highlighting potential cross-sectoral effects and trade-offs between regions.

EMPLOY builds on the FEMRIO framework from Wiebe, Bjelle [3] and adds interindustry dynamics by combining it with the non-linear input-output (NIO) model from Guerra and Sancho [4] and the Perhaps Adequate Demand System (PADS) from Almon [5]. Given the limited availability of consistent time-series for input-output tables (IOT) at state-level for the U.S., the model was designed for limited information conditions utilizing a generalized maximum entropy (GME) estimator for calibration.

In a traditional linear input-output model, intermediate input purchases from sector i by sector j (z_{ij}) are calculated as a linear function of total output in sector j (x_j) and the input coefficient α_{ij} (i.e., dollars of purchases from sector i per dollar of output in sector j), thus representing constant returns to scale:

$$z_{ij}(x_j) = \alpha_{ij} x_j$$

The NIO model allows for non-constant economies of scale in the production function of any given sector. Hence, each interindustrial flow between sectors (z_{ij}) is modeled as a

nonlinear function of the sector's output (x_j), which allows constant ($\beta_{ij} = 1$), increasing ($\beta_{ij} < 1$) or decreasing ($\beta_{ij} > 1$) returns to scale to intermediate input z_{ij}:

$$z_{ij}(x_j) = \alpha_{ij} x_j^{\beta_{ij}}; \alpha_{ij}, \beta_{ij} \geq 0$$

As previously mentioned, due to the limited availability of long time-series of harmonized input-output tables, the NIO model is estimated using a generalized maximum entropy (GME) estimator following Fernandez-Vazquez [6]:

$$\Delta \ln (z_{ijt}) = \beta_{ij} \Delta \ln (x_{jt}) + \varepsilon_{ijt} \quad t = 1, \ldots, T$$

The result from the maximum entropy program allows the recovery of both $\hat{\beta}_{ij}$ and $\hat{\alpha}_{ij}$. Hence, for each coefficient of the new nonlinear local input requirement matrix ($\mathbf{A}^*(\mathbf{x})$) we have:

$$a_{ij}(x_j) = \frac{z_{ij}(x_j)}{x_j} = \frac{\hat{\alpha}_{ij} x_j^{\hat{\beta}_{ij}}}{x_j} = \hat{\alpha}_{ij} x_j^{(\hat{\beta}_{ij} - 1)}$$

Based on the same NIO model, value added equations ($v_i(x_i)$) for each industry were estimated to reflect non-constant returns to scale:

$$v_i(x_i) = \alpha_i^{\mathrm{v}} x_i^{\beta_i^{\mathrm{v}}}$$

Given a total final demand vector ($\mathbf{f}$), the total output ($\mathbf{x}^*$) is obtained by solving the system of equations comprised of the demand and price models, where p are shadow prices:

$$\sum_i p_i z_{ij}(x_j) + p_v v_j(x_j) = \sum_i p_j z_{ji} (x_i) + p_j f_j = p_j x_j \qquad \forall j = 1, \ldots, n$$

The model uses 2017 environmental coefficients ($\mathbf{\Lambda}$) and related LCA factors ($\mathbf{F}$) to estimate several impact categories ($\mathbf{e}$) for each year. Those are directly applied to the total output ($\mathbf{x}^*$) solution from the non-linear IO model:

$$\mathbf{e} = \mathbf{F}' \mathbf{\Lambda} \mathbf{x}^*$$

Similarly, 2019 occupation-by-sector coefficients ($\mathbf{\Omega}$) are applied to the total output ($\mathbf{x}^*$) solution from the non-linear IO model:

$$\mathbf{O} = \mathbf{\Omega} \hat{\mathbf{x}}^*$$

Due to data limitations, both direct environmental and occupation coefficients are assumed to be constant across years.

EMPLOY also implements a household consumption model called the Perhaps Adequate Demand System (PADS) based on Almon [5], that accounts for price and

income effects. Personal consumption expenditure data from the U.S. Bureau of Economic Analysis (BEA) for each state are used to estimate the PADS based on the same GME estimator as before. The PADS is used during the calibration process of the overall model, but for this study it doesn't play a role in the simulations.

The base IOT time-series for calibration comprised of the State Make and Use Tables from the U.S. Environmental Protection Agency' (EPA) StateIO dataset [7] for 2012-2019. These are disaggregated into 71 sectors and 73 commodities for all states and the District of Columbia. Jobs data were derived from the BEA's Regional Accounts for 2012-2019. Satellite Accounts (releases to air, water and soil, resource use) were adapted from the 2017 State-Level Environmental Accounts from Avelino, Lamers [8]. Occupation matrices were estimated for 2019 based on multiple datasets from the U.S. Bureau of Labor Statistics' (BLS) Occupational Employment and Wage Statistics and O*Net Resource Center. The business-as-usual (BAU) economic baseline for the model uses real regional gross domestic product (GDP) and employment by industry data from Woods & Poole Economics [9] for 2020-2050. The model's base year is 2019 with 2012 constant prices (since the EPA tables are based off the 2012 National Benchmark IOTs from BEA).

For this study, we expanded the model's original disaggregation to 79 sectors and 88 commodities. These include existing industries that were disaggregated and the addition of new recycling sectors and commodities.

To better represent the glass industry, the glass manufacturing sector (NAICS 327210) was disaggregated in the 2012 Benchmark IOT using data from the 2002 Benchmark IOT [10], when the glass sector was separated according to Supplementary Figure 3 below. Due to the maturity of the industry, we assumed the 2002 production structure to proxy well the sector in 2012. We first mapped NAICS 2002 to NAICS 2012 classification, deflated the production structure to 2012 dollars using industry output deflators from BEA and then scaled the production in each sector based on the glass industry revenue data from Statista [11] for 2012. Finally, we balanced all flows to match the 2012 aggregated ones using a conventional RAS bi-proportional adjustment technique [12].

Additionally, we also disaggregated a few mineral and chemical commodities to more precisely model changes in raw material consumption for glass production when the share of cullet changes in the scenarios. Using data from the Mineral Commodity Summaries [13], we disaggregated silica and limestone from the "other non-metallic mineral mining and quarrying" commodity and soda ash from "other basic inorganic chemical" commodity (Supplementary Figure 3).

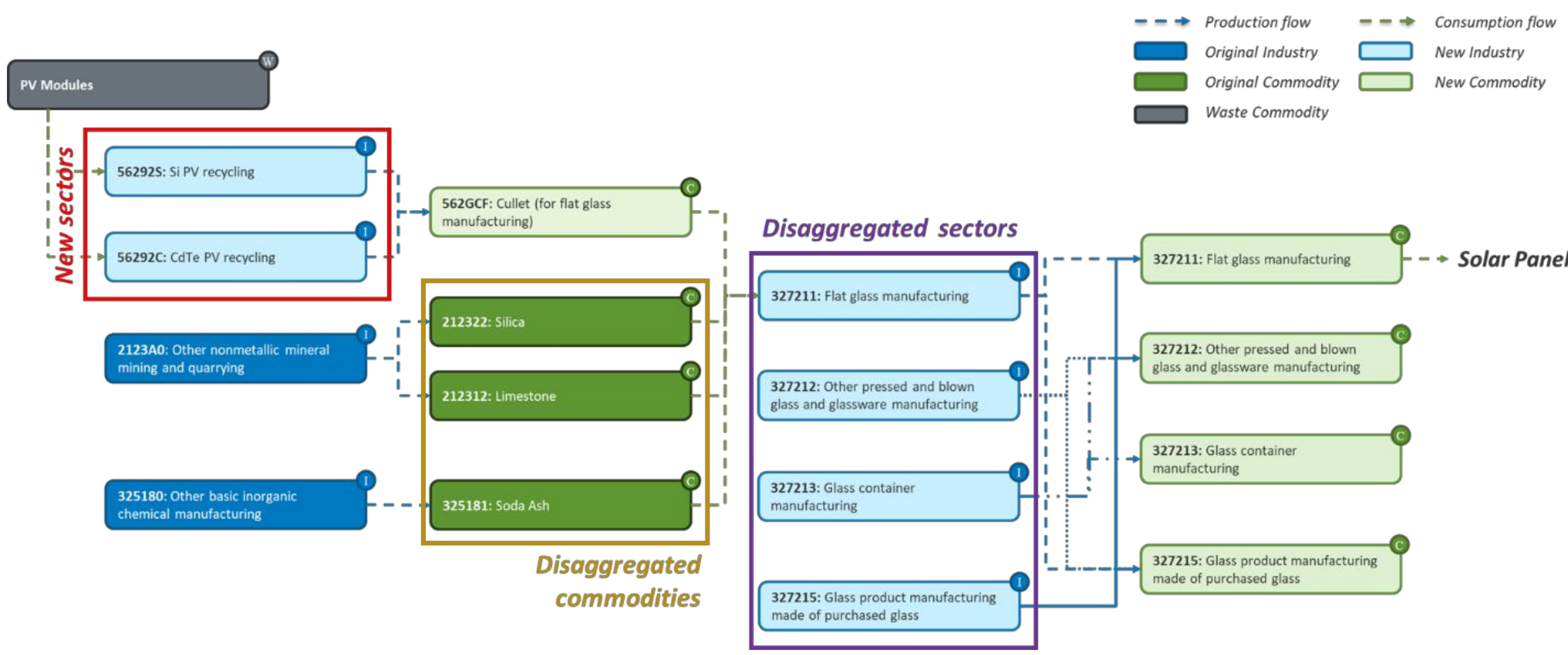

Supplementary Figure 3: Glass recycling and flat glass supply-chains and commodity outputs for the expanded EMPLOY model

We added two new PV recycling sectors to the model: 56292S – Silicon (Si) PV Recycling Industry and 56292C – Cadmium Telluride (CdTe) PV Recycling Industry. For sector 56292S (Si PV recycling), the engineering process was based on Vellini, Gambini [14] and Maani, Celik [15], with costs and labor requirements based on Tialpi's facility in Biella, Italy [16], the FRELP project [17], and standard TEA assumptions. Prices for co-products were based on various sources. The modeled industry was assumed to have a CapEx of $9.2 million and OpEx of $1.04 million/yr, which translates into a calculated cullet price of around $73/metric ton. Besides cullet, this industry produces other co-products as shown in Supplementary Figure 4. For sector 56292C (CdTe PV reclycling), the engineering process was also based on Vellini, Gambini [14] and Maani, Celik [15], with costs and labor requirements based on the Si PV Recycling Industry, and standard TEA assumptions. Prices for co-products were based on various sources. The modeled industry was assumed to have a CapEx of $9.2 million and OpEx of $0.74 million/yr, which translates into a calculated cullet price of around $39/metric ton. Besides cullet, this industry produces other co-products as shown in Supplementary Figure 5.

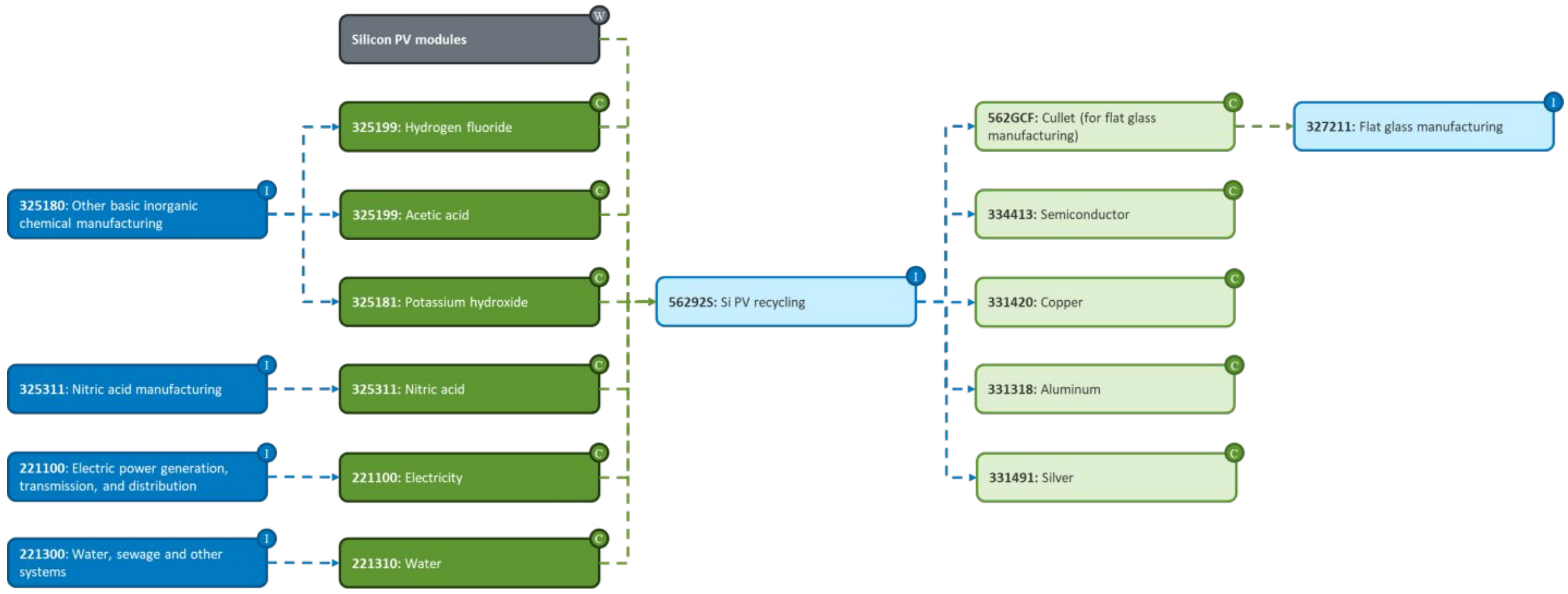

Supplementary Figure 4: Tier 1 supply-chains and commodity outputs for the Si PV recycling industry in the EMPLOY model

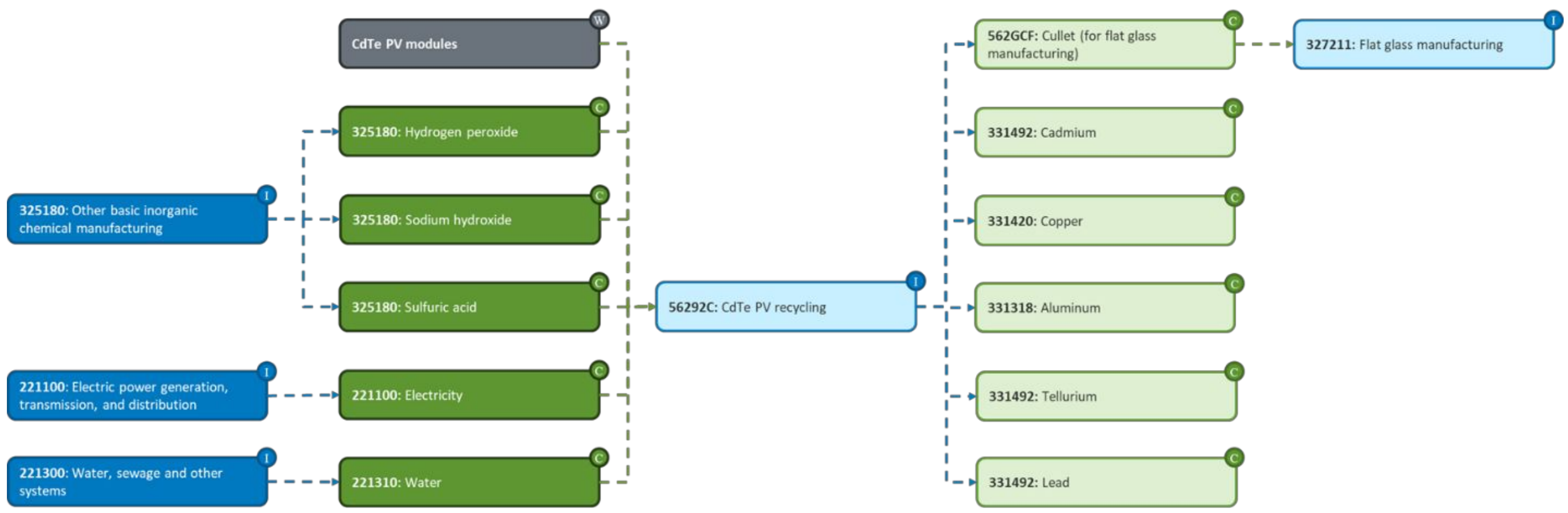

Supplementary Figure 5: Tier 1 supply-chains and commodity outputs for the CdTe PV recycling industry in the EMPLOY model

The sectors of interest for this study, in our case glass manufacturing and PV recycling, are exogenous, allowing us to generate different scenarios in terms of amount of cullet consumed versus raw materials and which PV pathway to use. The other sectors of the economy follow the non-linear dynamics described previously. Hence, the model runs according to the workflow in Supplementary Figure 6: for every year of the simulation, CELAVI provides EMPLOY with information on the amount of cullet and which PV recycling pathway to use. EMPLOY then reconfigures the production process for flat glass manufacturing by matching the cullet consumption and reducing the amounts of raw materials and energy required in the underlying Make and Use Tables. Similarly, the model scales the recycling pathway chosen and routes cullet sales to the flat glass manufacturing sector. Changes in power generation from the Regional Energy

Deployment System (ReEDS) model are captured in EMPLOY via a soft-link, allowing replicating the electricity mix from any ReEDS Standard Scenario by modifying the Make Table for the state. Once these changes are completed, the new adjusted IOT is passed back to the main model so it can run the NIO model and estimate jobs, sectoral output, value added and satellite accounts for that year according to the forecasted demand for flat glass. The process then repeats every subsequent year up to 2050. A BAU baseline is also estimated in EMPLOY, in which we use the same flat glass demand projection, but don't change the production structure of the flat glass manufacturing sector or recycling sectors. The net results are calculated from the difference between BAU and scenario simulation[1].

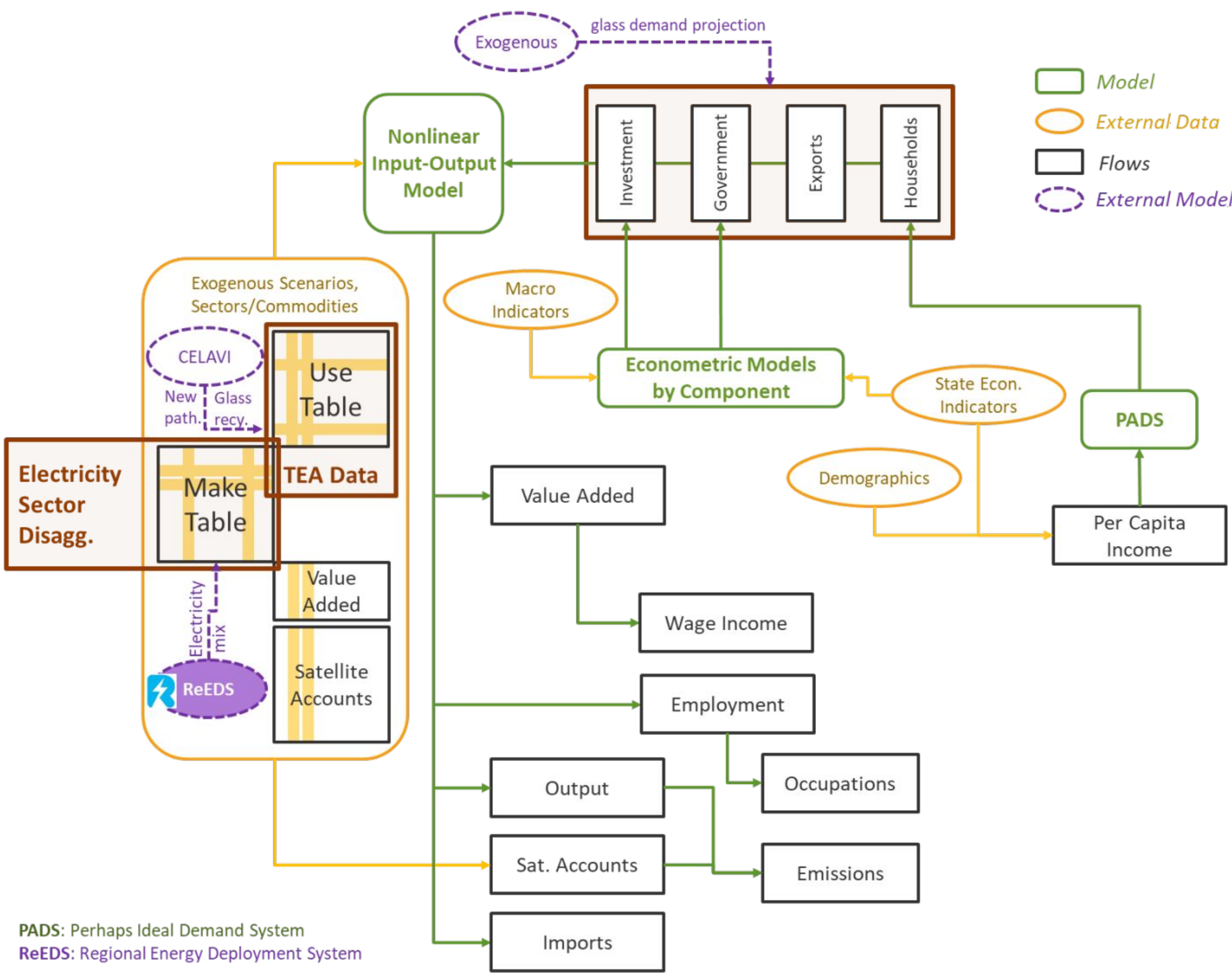

Supplementary Figure 6: Overview of the EMPLOY model workflow.

PIOCA Model Description:

[1] Note that because of the relative marginal impact the glass sector has on the electricity grid, there are no significant changes to be captured in ReEDS and so the power grid does not change between baseline and simulations.

PIOCA's main objective is: *"to quantify the effects of circularity on material flows at the national level."* The model uses IO tables from the U.S. Bureau of Economic Analysis (BEA, at the summary level, i.e., for 71 economic sectors) and with the exception of services, all economic flows within the BEA tables are converted into physical flows using data on trade and statistics from the U.S. Geological Survey (USGS). Similar to EMPLOY, the environmental satellite tables from EPA are used to evaluate the environmental impacts of circularity scenarios. Because the model has been applied to study decarbonization strategies within the iron & steel sector, that sector is already disaggregated [18]. Because CE strategies (such as lightweighting) can modify physical flows in the economy without changing monetary flows (or at least not in the same proportions), the model is better adapted to circularity analysis than monetary-based IO.

PIOCA was built using the following steps: 1) Calculate price vector from Comtrade data; 2) Calculate prices for primary extractive sectors from production data; 3) Compile complete price vectors; 4) Build supply and use tables in mass units; 5) Build monetary IO tables; 6) Build physical (mass) IO tables; 7) Calculate raw materials and residuals; 8) Balancing; 9) Produce final tables.

The construction of physical input output tables (PIOT) requires a price vector [19], but no such vector is available for the United States. UN Comtrade data documents both total mass and dollar values for imports and exports [20], so can be used for the calculation of a price vector. Initial estimates can be constructed via the use of import prices, due to the high use of imports in the domestic economy; for the supply table, export prices are often used [21]. In the case of the US economy, however, since exports make up a small portion of most production import prices were used where possible. Comtrade data is classified according to the Harmonized System (HS). A crosswalk to the BEA categories is used. A weighted average of prices is then derived according to the BEA categories. For zero entries not in the service or construction sectors, estimates are made according to the most similar groupings of NAICS codes. For example, if five digits of the NAICS code correspond, an average of those prices is used. Otherwise, four-digit matching by NAICS code is used, going down to the lowest number of digits at which a match is found.

Data sources for mining were evaluated following procedures detailed in EXIOBASE 3 construction [22]. Production data is converted into metric tons in the case of petroleum and natural gas, where volumetric measures are provided. There are six mining categories in the BEA output data, so mining production data is likewise aggregated to derive nominal annual prices at the detail and summary level. Official figures for total production available in mass units for extractive sectors were used directly to calculate prices based on the gross output values in the BEA tables by summing the output for each BEA category. The price vector was converted into the inverse price matrix, and this was used to construct use and make matrices in units of total mass.

Sectors whose goods are not imported or exported but whose goods can be measured in mass include construction, retail and wholesale, and restaurants. These sectors take in processed goods and resell them, so their output can be measured as a physical good. Other sectors are more explicitly services, such as legal services and

management. While these sectors take in physical products, we assume that these goods either become waste (consumables), capital stock (durable goods) or are combusted (fuels).

Construction, retail and wholesale trade, publishing, and food services can be assumed to have a physical product. For these sectors, the total inputs were summed and a price was estimated by the quotient of the total output in dollars for the sector and total inputs [21], as in the following equation:

$$p_{construction} = \frac{x_{construction}}{\sum_{j=1}^{n} z_{construction,\, j}}$$

PIOT also require the raw materials and waste flows that contribute to the overall commodity mass balance. For wastes, we relied on the total impact matrix as well as the total impact from domestic activity matrix from the US EEIO [23]. They act as satellite tables and were constructed for the year 2012, with units in kg/USD. They are integrated into the table by the following equation:

$$Nx = R{,}W$$

In this case, N refers to the total impact matrix, x corresponds to the total output vector, and the total impacts are segmented into raw materials and wastes. For this work the vectors for Commercial Construction and Demolition Debris, Commercial Municipal Solid Waste, Commercial RCRA Hazardous Waste and Pesticides are included in the Waste matrix.

CELAVI-EMPLOY-PIOCA linkage:

Finally, CELAVI material flows in the different landfilling and material recovery pathways of the supply chain are used as the IO models'. To do so, at each time step t CELAVI material flows m_{ij}^{t} are summed across all facilities i within each pathway types j according to the following equation:

$$m_j^t = \sum_i m_{ij}^t$$

For each pathway, m_j^t is inserted into it corresponding IO sector after being converted into monetary units. Those converted material flows are then used by EMPLOY to calculate the new state of the economy (using its technical coefficient matrix $\boldsymbol{A}$, final output vector $\boldsymbol{X}$, and scaled flow between economic sector $\boldsymbol{Z} = \boldsymbol{A} \cdot diag(\boldsymbol{X})$) and its related economy-wide socio-economic impacts. PIOCA converts the $\boldsymbol{X}$ vector and $\boldsymbol{Z}$ matrix back into physical units to provide a material perspective on the entire economy (see equation below).

$$\boldsymbol{Z_{\varphi}} = diag(\boldsymbol{P}^{-1}) \cdot \boldsymbol{Z} = diag(\boldsymbol{P}^{-1}) \cdot \boldsymbol{A} \cdot diag(\boldsymbol{X})$$

With $\boldsymbol{Z_{\varphi}}$ the matrix of scaled physical flows between economic sectors, $\boldsymbol{P}$ the PIOCA price vector, $\boldsymbol{Z}$ the matrix of scaled monetary flows between economic sectors and $\boldsymbol{A}$ and $\boldsymbol{X}$ as defined above.

These steps are repeated for each time step t of a particular scenario and different scenarios are explored through this method to provide SCORPIO's economy-wide insights into material recovery benefits and trade-offs. The script implementing the methodology connecting PIOCA and EMPLOY's data is available on a public repository:

https://github.com/NatLabRockies/SCORPIO.

2. Additional scenario details

Figure 1 in the main manuscript presents the different pathways modeled in SCORPIO. As the figure shows, components at EOL may travel along different pathways:

- Linear – to landfill.
- Open loop – to glass wool manufacturing or to supplementary cementitious material (SCM) production.
- Closed loop – to another lifetime as the same component.

The pathway choice is determined by the cost of accomplishing one closed loop or reaching the end of the supply chain.

Baseline:

The baseline cost scenario intends to model the current state of the glass sector. Data used to parameterize the different pathways (e.g., costs, losses) are collected from the literature and described in the supplementary table 1 below:

Supplementary Table 1: parameters and their values in the baseline (all instances of "tonne" in this supplementary information refer to metric tonne).

Parameter	Description	Value	Source
C_{uninst}^{pv}	Cost of decommissioning photovoltaics (PV) panels	\$300/kW	[24]
C_{dis}^{pv}	Cost of disassembling and recovering materials from PV panels	\$955-1910/tonne	[25, 26]
$L_{dis\&recov}^{pv}$	Learning rate for PV recycling	0.05	[2]
$Loss_{dis\&recov}^{pv}$	Losses from recycling process	2%-15%	[27]
$C_{remov\&recov}^{w}$	Cost of removing and handling windows	\$2800/tonne	[26, 28-30]
$L_{remov\&recov}^{w}$	Learning rate for window disassembly and glass recovery	0	Assumes no significant learning-by-doing from glaziers (already mature process & skills)
$C_{remov\&recov}^{w}$	Cost of refurbishing windows	\$8000-34000/tonne	[31, 32]
$L_{refurbish}^{w}$	Learning rate for window refurbishment	0	Assumes no significant learning-by-doing from glaziers (already mature process & skills)
$Loss_{remov\&recov}^{w}$	Losses from handling windows	0.6%	[33]

Parameter	Description	Value	Source
$Loss_{refurbish}^{w}$	Losses from refurbishing windows	3%	[34]
C_{cullet}^{w}	Flat glass beneficiation costs	$24-36/tonne	[26]
$L_{remov\&recov}^{w_{glass}}$	Contrary to windows reuse, losses from handling window glass are zero (no need to avoid breakage)	0	Assumption based on [33]
C_{cullet}	Cost of manufacturing cullet	$24-36/tonne	[26]
L_{cullet}	Learning rate for cullet manufacturing	0	Assumes no significant learning-by-doing from cullet manufacturing (already mature process & skills).
$Loss_{cullet}$	Losses from cullet manufacturing	0.6-5%	[26]
$C_{pozzolan}$	Cost for the additional steps from cullet to pozzolan manufacturing = ball mill, trommel screen, air classifier	$58-87/tonne	[26, 35]
$L_{pozzolan}$	Learning rate for pozzolan manufacturing	0	Assumes same learning as for cullet manufacturing
$Loss_{pozzolan}$	Losses from pozzolan manufacturing	0.6-5%	Assumes same losses as for cullet manufacturing
C_{land}	Landfill costs	$33-134/tonne	[36]
T	Transportation costs	$0.097/tonne.km	[37]
$dist$	Transportation distance (km)	Endogenous variable	[2]

Additional assumptions are:

- Lifetime assumptions: 20 years for PV panels [38] and 30 years for windows [39]; both are fixed values.
- Material compositions (kg glass per installed kW PV module and kg glass per m^2 commercial window): taken from Ovaitt, Mirletz [38] and Parker, Horsey [40] for PV and windows, respectively.
- The equation used to assign pathway costs in SCORPIO is:
$$Pathway\ costs = C_{pathway} \times L_{pathway} + Loss_{pathway} \times C_{landfill} + T \times dist$$
Note that some pathways involve multiple losses that are cumulative.
- The reuse of glass panes (window recovery and refurbishment) requires 100 person-hours/tonne of flat glass (with a crew of 2 person-hours per window) for the recovery and 100 person-hours/tonne of flat glass for the refurbishment ([26], goodwindowworks.com). The occupation for this process is glazier.
- The environmental impacts of removal and installation of the windows are neglected ([26], goodwindowworks.com).

- We assume that the glass beneficiation plant is similar to a material recovery facility (MRF). A similar method to Singh, Rorrer [41] is used to model flat glass recycling (see supplementary file 2).
- Growth rate for the glass manufacturing sector:
    - 3.00% from 2020 to 2050, excluding solar. (https://www.nrel.gov/docs/fy23osti/83730.pdf, page 17).
    - 4.08% from 2026 to 2050, when including all solar technologies (calculations based on the Regional Energy Deployment System (ReEDS) projections [42], Ovaitt, Mirletz [38], United Nations (UN) comtrade prices [20].
- When revenues are included in CELAVI's simulations (see Supplementary Table 6 and section 3.2 of the main manuscript), the following sources and assumptions are used as baseline:
    - For all supply chain process apart from "solar glass manufacturing from cullet", "module disassembly", "window glass recovery", "flat glass cullet manufacturing", "glass wool manufacturing", and "SCM manufacturing", a 10% net profit margin is assumed, in line with typical values in industry [43]. Hence, revenues were calculated as 110% of the gross process cost.
    - For "solar glass manufacturing from cullet" a 43% margin is assumed (i.e., revenues equal to 143% of gross process cost) as solar glass prices are assumed to stay the same while virgin material and energy costs are lower (see Supplementary Table 3).
    - For "module disassembly", revenues are assumed to be 20% of gross process costs, based on a previous study [27].
    - For "window glass recovery", revenues are assumed to be 13% of gross process costs, based on industry websites [44, 45].
    - For "flat glass cullet manufacturing" (from both solar and windows), revenues are assumed to be 80% of gross process costs.
    - For "glass wool manufacturing", a 116% margin is assumed as glass wool prices are assumed to stay the same while virgin material and energy costs are lower (see Supplementary Table 3).
    - For "SCM manufacturing", a 108% margin is assumed as cement prices are assumed to stay the same while virgin material and energy costs are lower (see Supplementary Table 3).

In the sensitivity analysis (Figure 3 in the main text), and specific scenarios other than the baseline (e.g., solar glass closed-loop recycling scenario in the main text), costs are varied from their baseline values (Supplementary Table 1) as described in the main manuscript.

<u>LCA data:</u>

Supplementary Table 2 presents the LCA data used to compute environmental impacts of the flat glass forward and reverse supply chains.

Supplementary Table 2: LCA data used in the flat glass case study

Activities	Reference	Ecoinvent	Unit	Celavi Unit	Comments
glass wool manufacturing	kilogram	glass wool mat production with cullet only	kilogram	kg	
landfilling	kilogram	treatment of municipal solid waste, sanitary landfill	kilogram	kg	
module disassembly	kilograms of solar glass	module disassembly	unit	kg	
module installation	kilograms of solar glass	electric installation, 570 kWp photovoltaic plant, at plant	unit	unit	Needs the module size in kW
module manufacturing	kilograms of solar glass	photovoltaic panel production, multi-Si wafer	square meter	square meter	Solar glass is removed
module uninstallation	kilograms of solar glass				
SCM manufacturing	kilogram	limestone production, crushed, for mill	kilogram	kg	
solar glass cullet manufacturing	kilogram	glass cullets, recovered from c-Si PV module treatment	kilogram	kg	
solar glass manufacturing	kilogram	flat glass production, uncoated	kilogram	kg	
solar glass manufacturing from cullet	kilogram	solar glass manufacturing from cullet	kilogram	kg	38% less energy input. 30% of original emissions are process emissions which is also deleted.
solar glass recovery	kilogram	solar glass recovery	kilogram	kg	
window glass manufacturing	kilogram	flat glass production, uncoated	kilogram	kg	
window glass recovery	kilogram				
window installation					
window manufacturing	kilograms of window glass	window frame production, aluminium, U=1.6 W/m2K	square meter	square meter	
window uninstallation					
window glass cullet manufacturing	kilogram	treatment of waste glass from unsorted public collection, sorting	kilogram	kg	
Transportation	tonne - km	transport, freight, lorry >32 metric tonne, EURO6	tonne kilometer	tonne - km	

3. Additional Figures and Tables

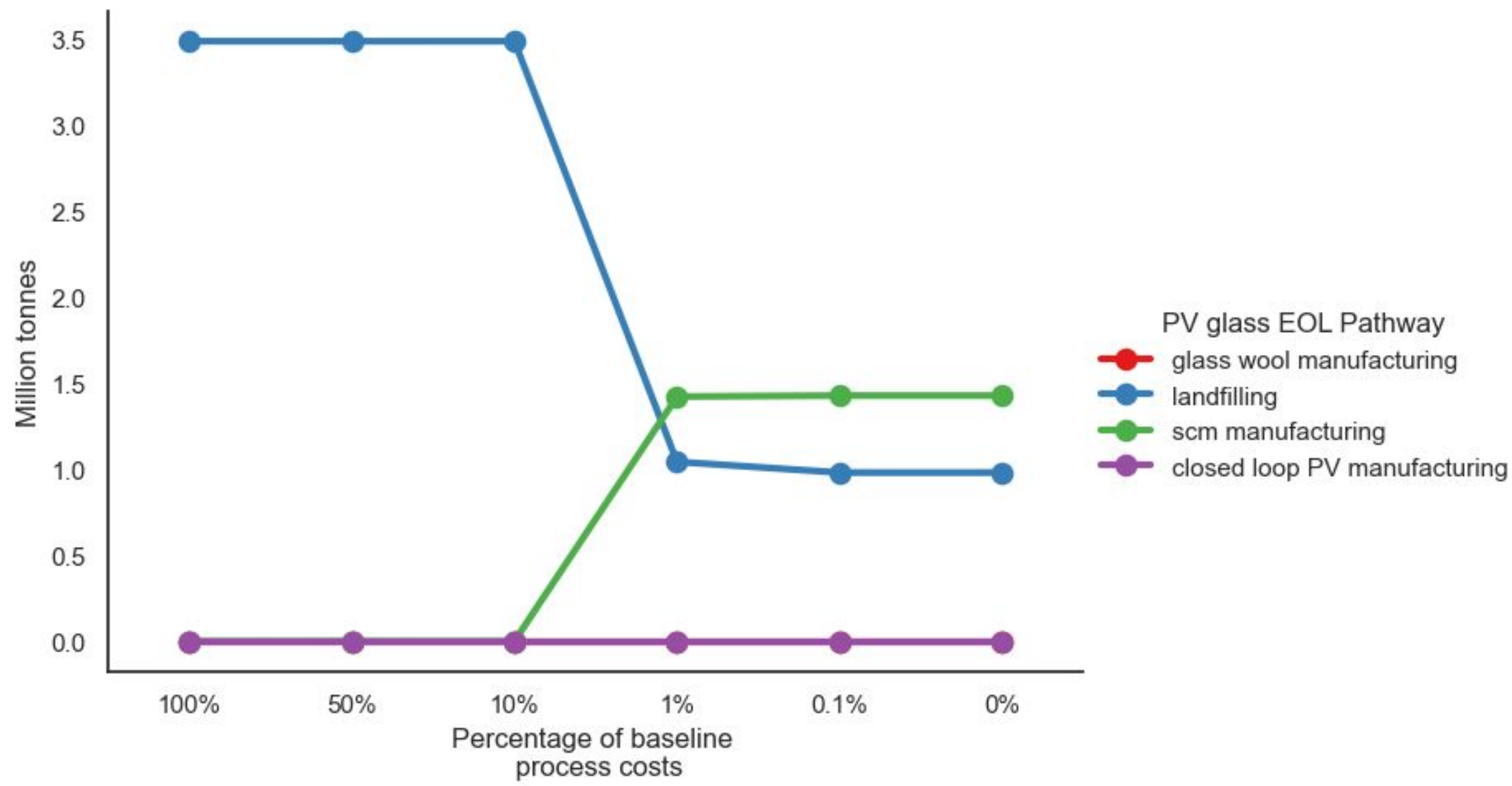

Supplementary Figure 7: Sensitivity analysis for six scenarios in which all PV glass recovery pathways compete. Mass flows in the different PV glass landfilling and material recovery pathways are given as a function of process cost values (from 100% to 0% of the baseline process costs) when the costs of **all four reverse supply chain processes** – PV module disassembly, closed-loop PV glass manufacturing from cullet, SCM manufacturing, and glass wool manufacturing – **are reduced simultaneously**.

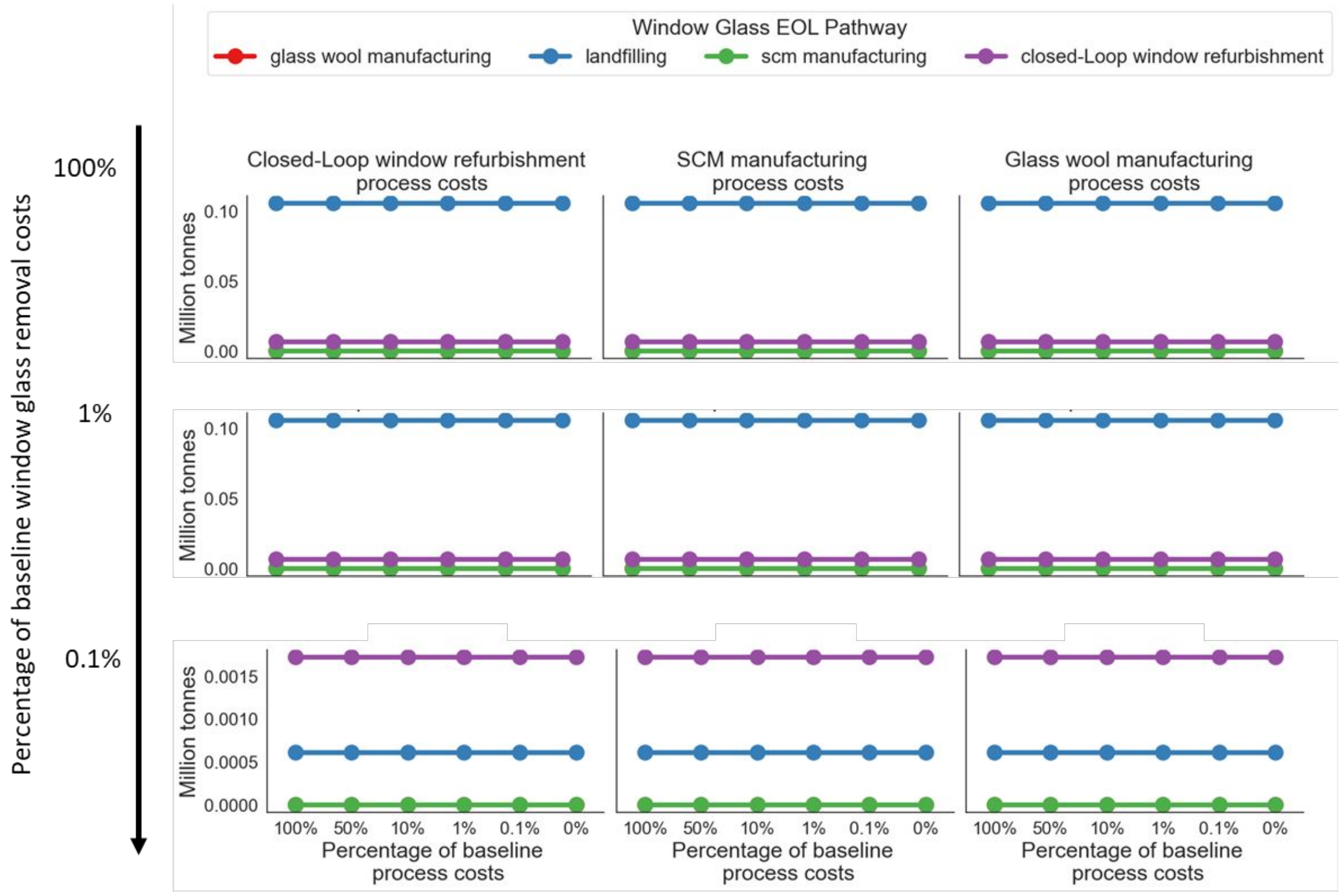

Supplementary Figure 8: Sensitivity analysis for 54 end-of-life scenarios for commercial building glass showing that window glass removal costs at 0.1% or below of their baseline enables closed loop window refurbishment (see Figure 1 of the main manuscript for the location of the different steps in the window glass supply chain). As seen in Figure 1 of the main manuscript, the landfilling, closed-loop recycling, and downcycling options compete with one another once windows are being decommissioned.

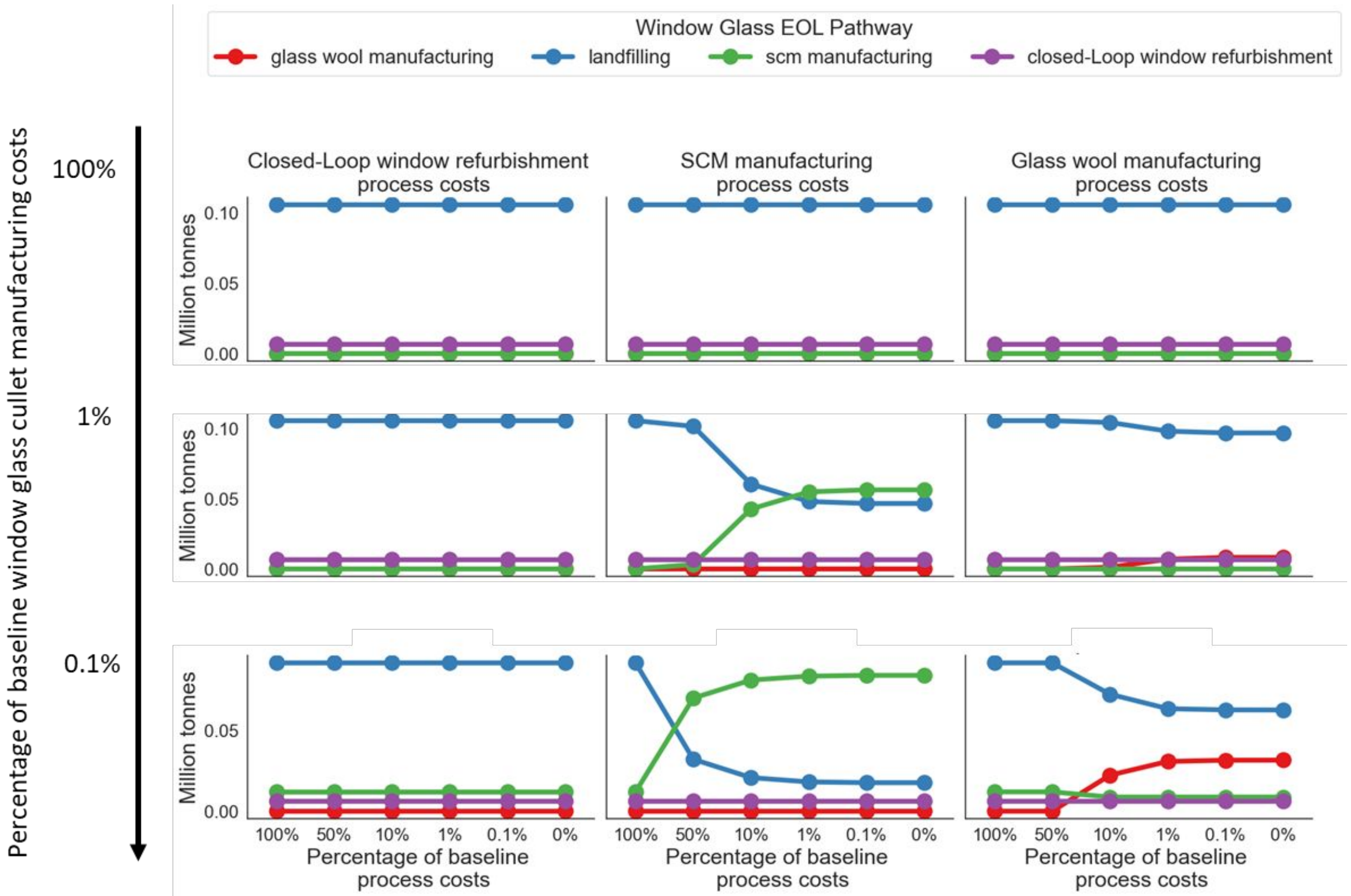

Supplementary Figure 9: Sensitivity analysis for 54 end-of-life scenarios for commercial building glass showing that window glass cullet manufacturing costs at 1% or below of their baseline in combination with supplementary cementitious material (SCM) manufacturing costs below 50% of their baseline or glass wool manufacturing costs below 10% of their baseline enables open loop recycling (either into glass wool or into SCM). As seen in Figure 1 of the main manuscript, the landfilling, closed-loop recycling, and downcycling options compete with one another once windows are being decommissioned.

Supplementary Table 3: unit costs, revenues, and net costs (in $/ton) for each of the reverse supply chain actors. Sources for unit cost and revenue are indicated in brackets. Unit costs are for the *Baseline* scenario*,* in the *PV-PV Closed-Loop* scenario, the solar recycler and flat glass manufacturer unit costs are 14.3 $/tonne and $7.0/tonne, respectively and in the *PV-SCM Open-Loop* scenario the solar recycler and SCM manufacturer unit costs are 14.3 $/tonne and $7.2/tonne (see Supplementary Figure 9).

**Supply chain actor**	**Unit Costs ($/tonne) [source]**	**Unit Revenues ($/tonne) [source]**	**Net unit cost($/tonne)**
Solar recycler (module disassembly, solar glass recovery)	1433 [25, 26]	285 [27]	**1148**
Glass beneficiation (solar glass cullet manufacturing)	30 [26]	54 [26]	**-24**
Flat glass manufacturer (solar glass manufacturing from cullet)	700 (assumes 10% margins, minus raw material costs estimated from USGS data [46])	1000 [47]	**-300**
Glass wool manufacturer (glass wool manufacturing)	220 (assumes 10% margins, minus raw material costs estimated from USGS data [46])	475 [48]	**-255**
SCM manufacturer (SCM manufacturing)	72 [26, 35]	150 [49]	**-78**
Landfill (landfilling)	30 [36]	0.0 [36]	**30**

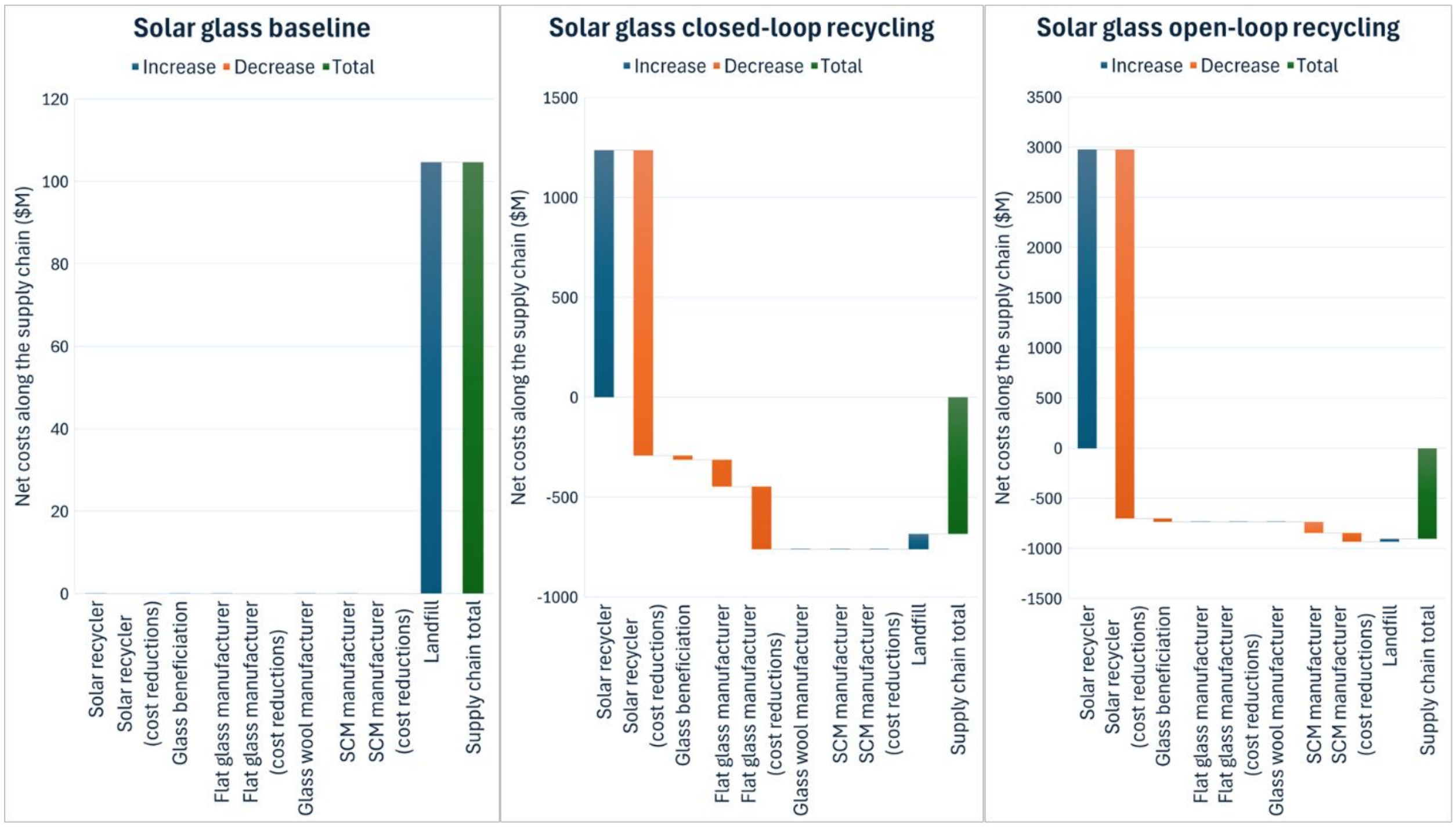

Supplementary Figure 10: Cumulative net costs (in million dollars ($ M)) for each of the reverse supply chain actors for the different scenarios over the 2025-2050 period, including cost reductions. Net negative values are shown in blue (i.e., net loss for the supply chain actor), while net positive values (i.e., net revenue or cost reductions) are shown in orange. Total cost and total revenues are calculated by multiplying the unit cost ($/ton) and unit revenue ($/ton) in Supplementary Table 3 by the total mass flow seen by each supply chain actor over the 2025-2050 period (in million metric tons (Mt)). Cost reductions are calculated according to each scenarios' assumptions and could stem from a combination of specific factors, including investments in recycling technologies, research and development (R&D) into material recovery processes, increased revenues from the sale of recovered materials, and policy mechanisms aimed at incentivizing recycling.

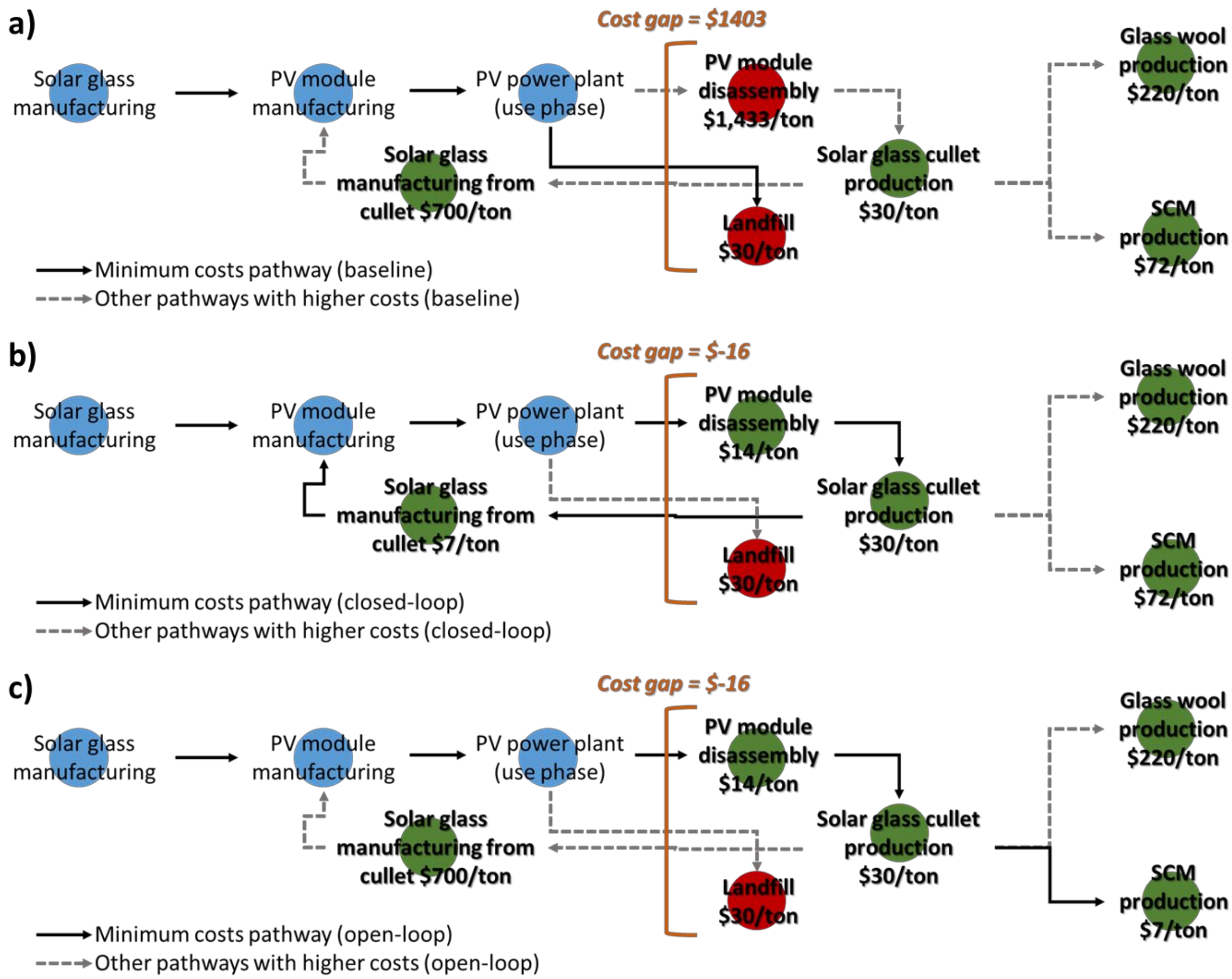

Supplementary Figure 11: Simplified diagram of the supply chain superstructure modeled in SCORPIO in a) the Baseline, b) PV-PV Closed-Loop recycling (module disassembly costs = 1% baseline and solar glass manufacturing from cullet costs = 1% baseline), and c) PV-SCM Open-Loop recycling (module disassembly costs = 1% baseline and SCM manufacturing costs = 10% baseline) scenarios. The reverse supply chain costs in the different scenarios are indicated below the names of each of the steps in the reverse supply chain. For each step, a green color node indicates a net revenue while a red color indicates a net cost (see Supplementary Table 3).

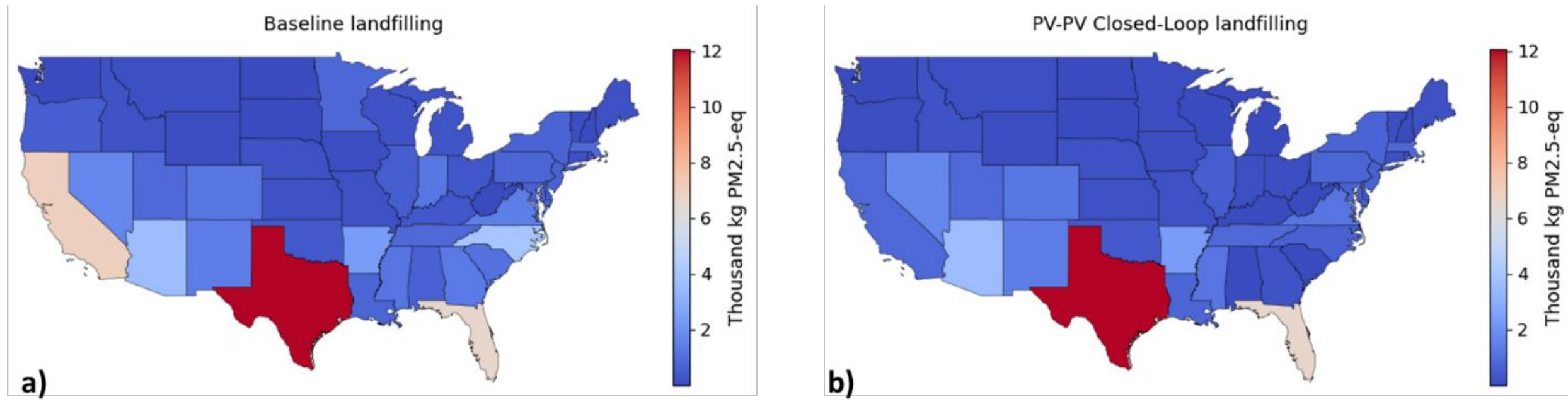

Supplementary Figure 12: Distribution of particulate matter emissions (respiratory effects category in TRACI) from U.S. landfill sites in a) the baseline and b) PV-PV closed-loop recycling scenarios.

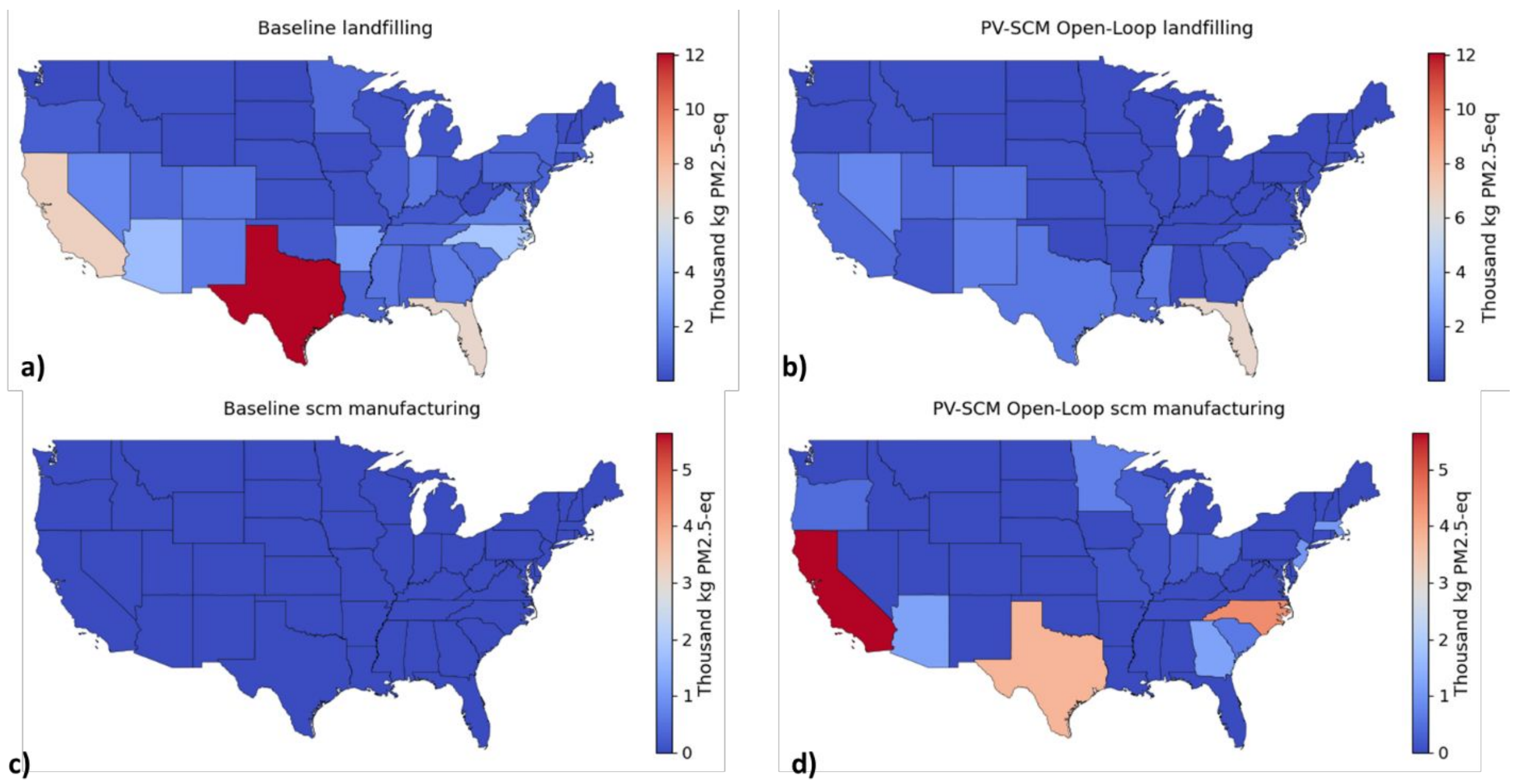

Supplementary Figure 13: Distribution of particulate matter emissions (respiratory effects category in TRACI) from U.S. landfill (a, b) and cement manufacturing sites (c, d) in the baseline (a, c) and PV-SCM open-loop recycling (b, d) scenarios.

Supplementary Table 4: life cycle assessment**:** LCA results for the Baseline, PV-PV Closed-Loop, and PV-SCM Open-Loop scenarios. Impact scores were calculated with the TRACI methodology [50]. In the solar glass closed-loop recycling, avoided impacts from the manufacturing of flat glass from raw materials (due to the manufacturing of flat glass from cullet) are already included in the net impact column. In the SCM open-loop recycling, avoided impacts from Portland cement manufacturing (due to the replacement of part of the Portland cement by SCM in concrete) are reported separately.

		**Baseline**	**Solar glass closed-loop recycling**	**SCM open-loop recycling**		
**Impact category**	**Unit**	**Impact**	**Net impact**	Impact	Avoided impact	**Net impact**
Acidification, moles of H+-Eq	moles of H+-Eq	**1.37E+08**	**1.36E+08**	1.37E+08	2.20E+06	**1.34E+08**
Carcinogenics, kg benzene-Eq	kg benzene-Eq	**7.54E+06**	**7.54E+06**	7.54E+06	2.68E+01	**7.54E+06**
Ecotoxicity, kg 2,4-D-Eq	kg 2,4-D-Eq	**4.87E+08**	**4.87E+08**	4.87E+08	4.63E+09	**-4.14E+09**
Eutrophication, kg N	kg N	**3.14E+05**	**3.13E+05**	3.14E+05	1.06E+06	**-7.43E+05**
Global Warming, kg CO2-Eq	kg CO2-Eq	**3.27E+08**	**3.26E+08**	3.27E+08	1.25E+09	**-9.22E+08**
Non-Carcinogenics, kg toluene-Eq	kg toluene-Eq	**9.38E+09**	**9.37E+09**	9.38E+09	1.48E+02	**9.38E+09**
Ozone Depletion, kg CFC-11-Eq	kg CFC-11-Eq	**2.62E+01**	**2.61E+01**	2.62E+01	3.21E+00	**2.29E+01**
Photochemical Oxidation, kg NOx-Eq	kg NOx-Eq	**1.01E+06**	**1.01E+06**	1.01E+06	4.67E+07	**-4.57E+07**
Respiratory Effects, Average, kg PM2.5-Eq	kg PM2.5-Eq	**6.18E+05**	**6.17E+05**	6.18E+05	3.07E+05	**3.11E+05**

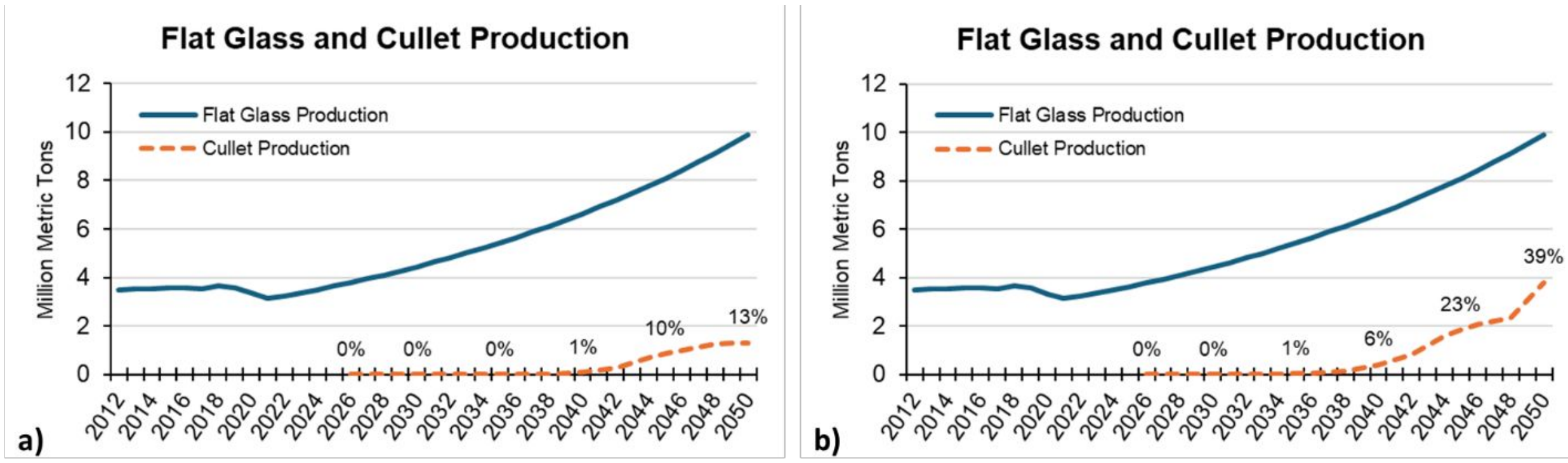

Supplementary Figure 14: Cullet production from crystalline Silicon (c-Si) PV flat glass recycling compared to total flat glass production: a) in the PV-PV Closed-Loop recycling scenario (module disassembly costs = 1% baseline and solar glass cullet manufacturing costs = 1% baseline), b) in the ideal 100% PV-PV recycling scenario where 100% of the PV panels are being recycled.

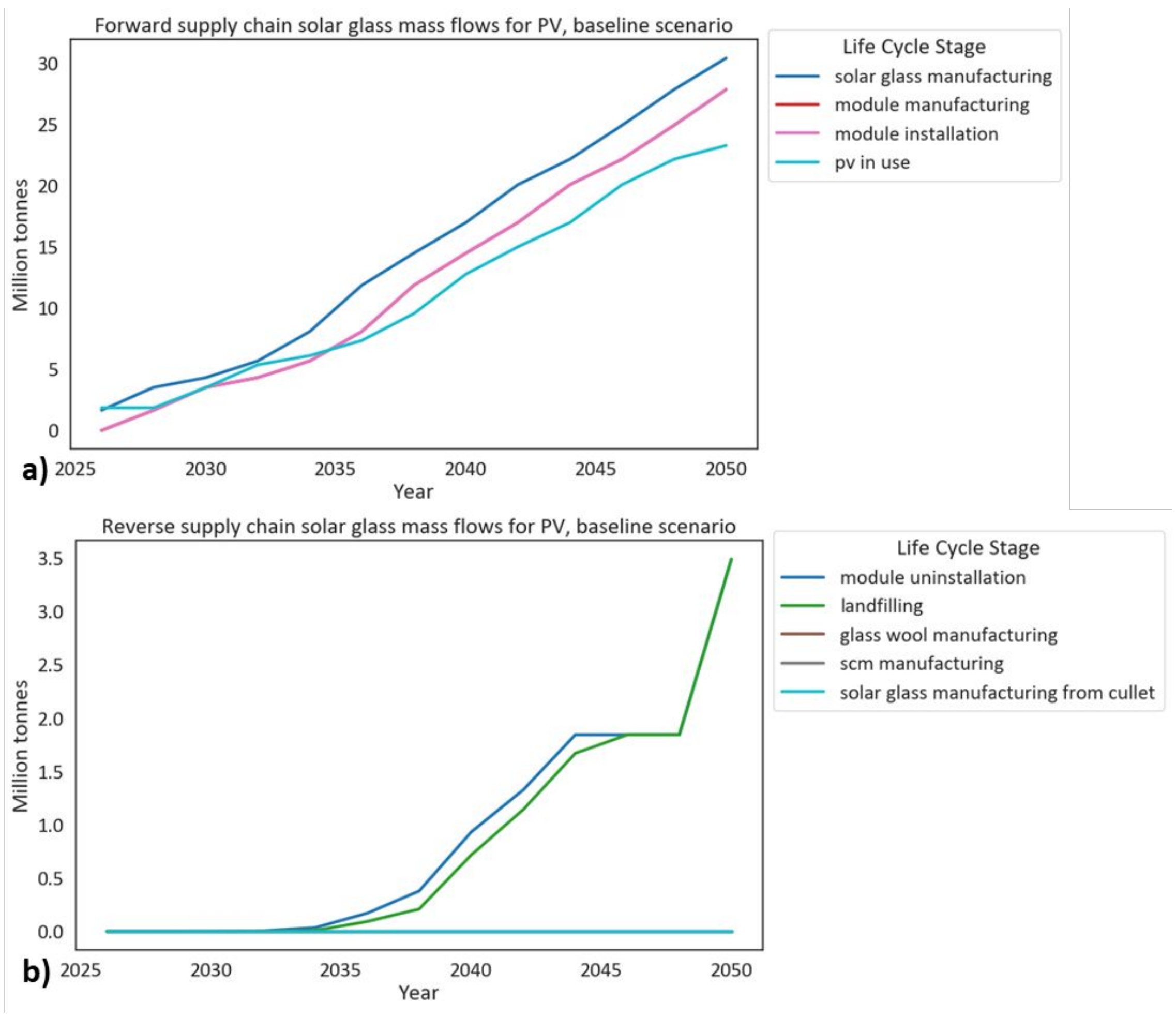

Supplementary Figure 15: Mass flows during the different stages of the forward and reverse PV supply chain in the baseline scenario.

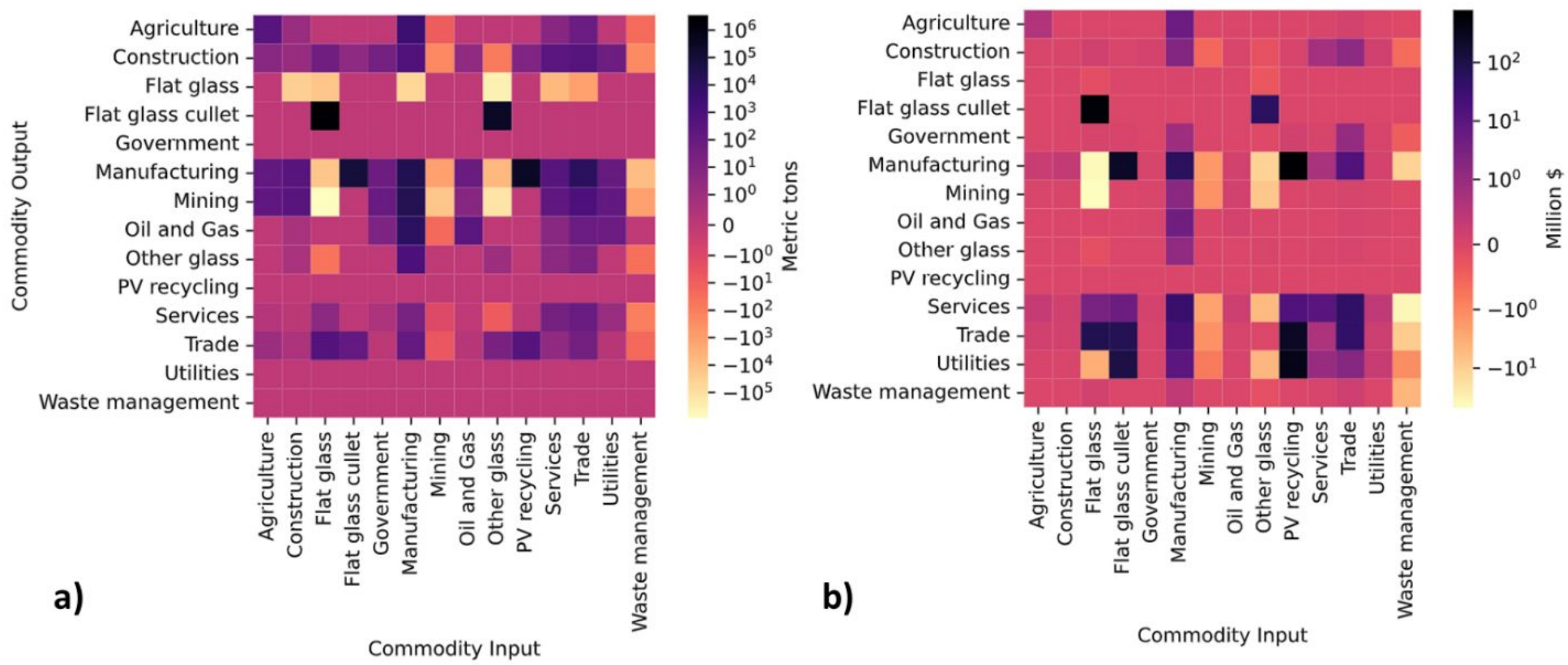

Supplementary Figure 16: Structural changes in the U.S. economy between the 100% PV-PV recycling scenario and baseline scenarios as reported by SCORPIO's a) physical IO tables, b) monetary IO tables. Note that economic sectors have been aggregated for clarity in this figure (Supplementary Table 5)

Supplementary Table 5: Sectoral aggregation for the heat maps in the main text Figure 7 and Supplementary Figure 15.

IO Code	Name	Custom grouping	Custom10 grouping
111CA	Farms	Agriculture	Agriculture
113FF	Forestry, fishing, and related activities	Agriculture	Agriculture
211	Oil and gas extraction	Oil and Gas	Oil and Gas
212	Mining, except oil and gas	Mining	Mining
213	Support activities for mining	Mining	Mining
22	Utilities	Utilities	Utilities
23	Construction	Construction	Construction
321	Wood products	Wood and paper	Manufacturing
327	Nonmetallic mineral products	Mineral products	Manufacturing
327211	Flat Glass	Glass: flat	Flat glass
327212	Pressed and blown glass	Glass: pressed and blown	Other glass
327213	Container glass	Glass: container	Other glass

IO Code	Name	Custom grouping	Custom10 grouping
327215	Glass products	Glass: products	Other glass
331	Primary metals	Metals and machinery	Manufacturing
332	Fabricated metal products	Metals and machinery	Manufacturing
333	Machinery	Machinery	Manufacturing
334	Computer and electronic products	Machinery	Manufacturing
335	Electrical equipment, appliances, and components	Machinery	Manufacturing
3361MV	Motor vehicles, bodies and trailers, and parts	Machinery	Manufacturing
3364OT	Other transportation equipment	Machinery	Manufacturing
337	Furniture and related products	Furniture	Manufacturing
339	Miscellaneous manufacturing	Other manufacturing	Manufacturing
311FT	Food and beverage and tobacco products	Consumables	Manufacturing
313TT	Textile mills and textile product mills	Apparel and textiles	Manufacturing
315AL	Apparel and leather and allied products	Apparel and textiles	Manufacturing
322	Paper products	Wood and paper	Manufacturing
323	Printing and related support activities	Wood and paper	Manufacturing
324	Petroleum and coal products	Petroleum and coal	Manufacturing
325	Chemical products	Chemicals and plastics	Manufacturing
326	Plastics and rubber products	Chemicals and plastics	Manufacturing
42	Wholesale trade	Wholesale Trade	Trade
441	Motor vehicle and parts dealers	Retail	Trade
445	Food and beverage stores	Retail	Trade
452	General merchandise stores	Retail	Trade
4A0	Other retail	Retail	Trade
481	Air transportation	Transport and storage	Trade

IO Code	Name	Custom grouping	Custom10 grouping
482	Rail transportation	Transport and storage	Trade
483	Water transportation	Transport and storage	Trade
484	Truck transportation	Transport and storage	Trade
485	Transit and ground passenger transportation	Transport and storage	Trade
486	Pipeline transportation	Transport and storage	Trade
487OS	Other transportation and support activities	Transport and storage	Trade
493	Warehousing and storage	Transport and storage	Trade
511	Publishing industries, except internet (includes software)	Professional and Educational Services	Services
512	Motion picture and sound recording industries	Professional and Educational Services	Services
513	Broadcasting and telecommunications	Professional and Educational Services	Services
514	Data processing, internet publishing, and other information services	Professional and Educational Services	Services
521CI	Federal Reserve banks, credit intermediation, and related activities	Professional and Educational Services	Services
523	Securities, commodity contracts, and investments	Professional and Educational Services	Services
524	Insurance carriers and related activities	Professional and Educational Services	Services
525	Funds, trusts, and other financial vehicles	Professional and Educational Services	Services
HS	Housing	Housing and Real Estate	Services
ORE	Other real estate	Housing and Real Estate	Services
532RL	Rental and leasing services and lessors of intangible assets	Housing and Real Estate	Services
5411	Legal services	Professional and Educational Services	Services
5415	Computer systems design and related services	Professional and Educational Services	Services
5412OP	Miscellaneous professional, scientific, and technical services	Professional and Educational Services	Services
55	Management of companies and enterprises	Professional and Educational Services	Services
561	Administrative and support services	Professional and Educational Services	Services

IO Code	Name	Custom grouping	Custom10 grouping
562	Waste management and remediation services	Waste management	Waste management
5629S0	Recycling 1	Recycling 1	Flat glass cullet
5629S1	Recycling 2	Recycling 2	PV recycling
5629S2	Recycling 3	Recycling 3	PV recycling
5629S3	Recycling 4	Recycling 4	PV recycling
5629S4	Recycling 5	Recycling 5	PV recycling
61	Educational services	Professional and Educational Services	Services
621	Ambulatory health care services	Health care	Services
622	Hospitals	Health care	Services
623	Nursing and residential care facilities	Health care	Services
624	Social assistance	Health care	Services
711AS	Performing arts, spectator sports, museums, and related activities	Leisure	Services
713	Amusements, gambling, and recreation industries	Leisure	Services
721	Accommodation	Leisure	Services
722	Food services and drinking places	Leisure	Services
81	Other services, except government	Other services	Services
GFGD	Federal general government (defense)	Government	Government
GFGN	Federal general government (nondefense)	Government	Government
GFE	Federal government enterprises	Government	Government
GSLG	State and local general government	Government	Government
GSLE	State and local government enterprises	Government	Government

A basic sensitivity analysis was performed on revenue from processes within the PV and window flat glass reverse supply chain. All process costs, for both forward and reverse supply chain processes, were kept at baseline levels throughout this analysis, and process revenues for processes in the forward supply chain likewise were kept at baseline levels (zero). No change in cumulative landfilled material (million metric tonnes) was observed relative to the baseline scenario when process revenues were included in the simulation.

Supplementary Table 6: Results of the sensitivity analysis performed on reverse supply chain process revenues.

**Process Costs (forward and reverse supply chain)**		100%	100%	100%	100%
**Process Revenues (reverse supply chain)**		0%	100%	25%	175%
**Process Revenues (forward supply chain)**		0%	0%	0%	0%
**Reverse Supply Chain Processes**		**Cumulative Landfilled Material, 2050 (million metric tonnes)**			
**PV**	**module uninstallation**	3.5	3.5	3.5	3.5
	**landfilling**	3.5	3.5	3.5	3.5
	**glass wool manufacturing**	0	0	0	0
	**scm manufacturing**	0	0	0	0
	**solar glass manufacturing from cullet**	0	0	0	0
**Window**	**window uninstallation**	0.11	0.11	0.11	0.11
	**landfilling**	0.10	0.10	0.10	0.10
	**glass wool manufacturing**	0	0	0	0
	**scm manufacturing**	0	0	0	0
	**window glass cullet manufacturing**	0	0	0	0
	**window glass recovery**	0	0	0	0

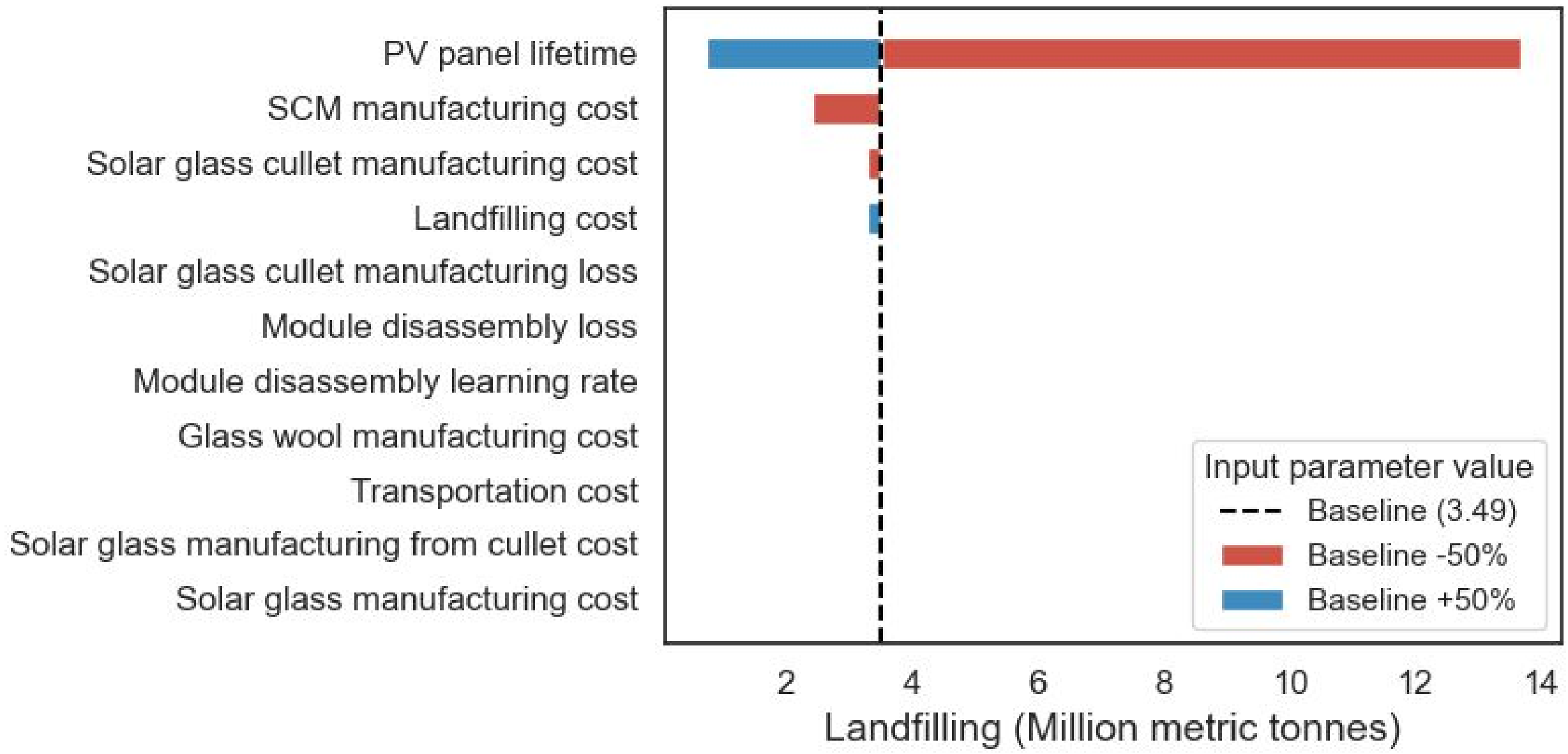

Supplementary Figure 17: Overall sensitivity analysis of the PV reverse supply chain, beyond cost parameters. For all scenarios, PV module disassembly costs are set to zero (to remove this additional circularity bottleneck which was identified in Figure 3 cost sensitivity analysis).

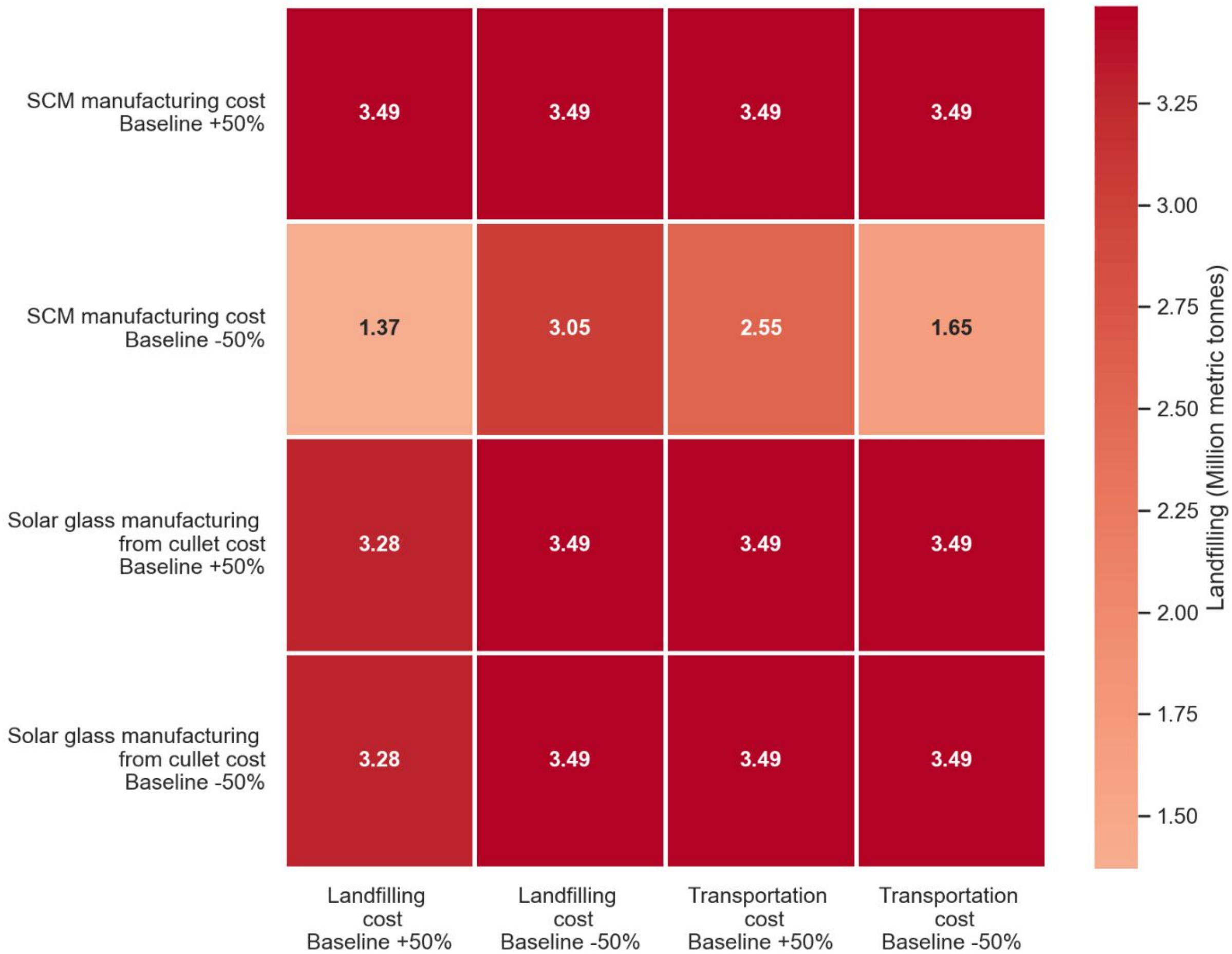

Supplementary Figure 18: $(2x2)^2$ sensitivity analysis where two main parameters – the ones varied in the PV-PV Closed-Loop scenario and PV-SCM Open-Loop scenario – and two secondary cost parameters (chosen based on Supplementary Figure 17 results) are varied together to their baseline value -50% or their baseline value +50%.

4. References

1. Walzberg, J., et al., *The inclusion of uncertainty in circularity transition modeling: A case study on wind turbine blade end-of-life management.* Sustainable Energy Technologies and Assessments, 2023. **60**: p. 103569.
2. Hanes, R., et al., *The Circular Economy Lifecycle Assessment and Visualization Framework: A Case Study of Wind Blade Circularity in Texas.* Frontiers in Sustainability, 2021. **2**(45).
3. Wiebe, K.S., et al., *Implementing exogenous scenarios in a global MRIO model for the estimation of future environmental footprints.* Journal of Economic Structures, 2018. **7**(1): p. 20.
4. Guerra, A.-I. and F. Sancho, *An operational, nonlinear input–output system.* Economic Modelling, 2014. **41**: p. 99-108.
5. Almon, C., *The craft of economic modeling*. 2017: www. CreateSpace. com.
6. Fernandez-Vazquez, E., *EMPIRICAL ESTIMATION OF NON-LINEAR INPUT–OUTPUT MODELS: AN ENTROPY ECONOMETRICS APPROACH.* Economic Systems Research, 2015. **27**(4): p. 508-524.
7. Li, M., et al., *StateIO - Open Source Economic Input-Output Models for the 50 States of the United States of America.* International Regional Science Review, 2023. **46**(4): p. 428-481.
8. Avelino, A.F.T., et al., *Creating a harmonized time series of environmentally-extended input-output tables to assess the evolution of the US bioeconomy - A retrospective analysis of corn ethanol and soybean biodiesel.* Journal of Cleaner Production, 2021. **321**: p. 128890.
9. Woods & Poole Economics. *Regional gross domestic product and employment by industry*. 2023; Available from: https://www.woodsandpoole.com/.
10. BEA. *Bureau of Economic analysis - Input-Output Accounts Data* 2020 [cited 2020 5/5/2020]; Available from: https://www.bea.gov/.
11. Statista. *Industry revenue of "glass and glass product manufacturing" in the U.S. from 2012 to 2024*. 2024 [cited 2026 7/17/2026]; Available from: https://www.statista.com/forecasts/310900/glass-and-glass-product-manufacturing-revenue-in-the-us/?srsltid=AfmBOop1sqKTBL-a-PtgMULXcQoPiQt0lCdVDBSL4ymDtL2GQz2Bl8qb.
12. Miller, R.E. and P.D. Blair, *Input-output analysis: foundations and extensions*. 2009: Cambridge University Press.
13. USGS. *Silica Statistics and Information*. 2010-2022; Available from: https://www.usgs.gov/centers/national-minerals-information-center/silica-statistics-and-information.
14. Vellini, M., M. Gambini, and V. Prattella, *Environmental impacts of PV technology throughout the life cycle: Importance of the end-of-life management for Si-panels and CdTe-panels.* Energy, 2017. **138**: p. 1099-1111.
15. Maani, T., et al., *Environmental impacts of recycling crystalline silicon (c-SI) and cadmium telluride (CDTE) solar panels.* Science of The Total Environment, 2020. **735**: p. 138827.
16. Bellini, E. *New industrial plant concept for end-of-life PV panel recycling*. 2022 [cited 2026 7/17/2026]; Available from: https://www.pv-magazine.com/2022/10/24/new-industrial-plant-concept-for-end-of-life-pv-panel-recycling/.

17. Ramon, L., et al., *FRELP - Full Recovery End of Life Photovoltaic*. 2014.
18. Wachs, L. and C. McMillan. *Measuring Economy-Wide Circularity of the United States: An Input-Output Model in Mass Units.* . 2023.
19. Aguilar-Hernandez, G.A., et al., *Assessing circularity interventions: a review of EEIOA-based studies.* Journal of Economic Structures, 2018. **7**(1): p. 14.
20. United Nations. *UN Comtrade Database*. 2023 [cited 2023 01/10/2023]; Available from: comtrade.un.org.
21. Milletler, B., *Handbook on supply and use tables and input output-tables with extentions and applications*. 2018, New York: United Nations Publications.
22. Merciai, S. and J. Schmidt, *Physical/hybrid supply and use tables.* Methodological Report http://fp7desire. eu/documents/category/3-public-deliverables, EU FP7 DESIRE Project, 2016.
23. Yang, Y., et al., *USEEIO: A new and transparent United States environmentally-extended input-output model.* Journal of Cleaner Production, 2017. **158**: p. 308-318.
24. Curtis, T.L., et al., *Solar Photovoltaic Module Recycling: A Survey of US Policies and Initiatives.* 2021, National Renewable Energy Laboratory.: Golden, Colorado.
25. EPRI, *Solar PV module end of life: options and knowledge for utility-scale plants*. 2018.
26. Farel, R., et al., *A cost and benefit analysis of future end-of-life vehicle glazing recycling in France: A systematic approach.* Resources, Conservation and Recycling, 2013. **74**: p. 54-65.
27. Walzberg, J., A. Carpenter, and G.A. Heath, *Role of the social factors in success of solar photovoltaic reuse and recycle programmes.* Nature Energy, 2021.
28. Windowinnovations.com. *Marvin Window and Door Dealer in Omaha & Papillion, NE*. 2025 [cited 2025 05/05/2025]; Available from: Windowinnovations.com.
29. syracuseglass.com. *Syracuse Glass Company*. 2025 [cited 2025 05/05/2025]; Available from: syracuseglass.com.
30. architecturaldigest.com. *AD*. 2025 [cited 2025 05/05/2025].
31. CNN. *Cost of window repair*. 2025 [cited 2025 05/05/2025]; Available from: https://www.cnn.com/cnn-underscored/home/cost-of-window-repair#:~:text=Key%20takeaways,bay%2C%20bow%20and%20garden%20windows.
32. RetroFit. *Project Case Study: Empire State Building*. 2009 [cited 2025 05/05/2025]; Available from: https://rmi.org/wp-content/uploads/2017/04/Buildings_Retrofit_EmpireStateBuilding_CaseStudy_2009.pdf
33. Qurbanov, A.M. and M.J. Baxtiyorovich, *REDUCING GLASS LOSS DURING TRANSPORTATION AND STORAGE.* Technical science and innovation, 2024. **2024**(2): p. 76-82.
34. Kennett, S. *News Empire State Building window refurb: The empire strikes back*. 2010 [cited 2025 05/05/2025]; Available from: https://www.building.co.uk/news/empire-state-building-window-refurb-the-empire-strikes-back/5005104.article#:~:text=About%2097%25%20of%20the%20glass,an%20example%20others%20will%20follow.

35. Tucker, E.L., et al., *Economic and life cycle assessment of recycling municipal glass as a pozzolan in portland cement concrete production.* Resources, Conservation and Recycling, 2018. **129**: p. 240-247.
36. EREF, *Analysis of MSW Landfill Tipping Fees*. 2023.
37. Iloeje, C.O., et al., *A systematic analysis of the costs and environmental impacts of critical materials recovery from hybrid electric vehicle batteries in the U.S.* iScience, 2022. **25**(9): p. 104830.
38. Ovaitt, S., et al., *PV in the circular economy, a dynamic framework analyzing technology evolution and reliability impacts.* iScience, 2022. **25**(1): p. 103488.
39. Citherlet, S., F. Di Guglielmo, and J.-B. Gay, *Window and advanced glazing systems life cycle assessment.* Energy and Buildings, 2000. **32**(3): p. 225-234.
40. Parker, A., et al., *ComStock Reference Documentation*. 2025, National Renewable Energy Laboratory: Golden, CO.
41. Singh, A., et al., *Techno-economic, life-cycle, and socioeconomic impact analysis of enzymatic recycling of poly(ethylene terephthalate).* Joule, 2021. **5**(9): p. 2479-2503.
42. Ho, J., et al., *Regional Energy Deployment System (ReEDS) Model Documentation (Version 2020)*. 2021: United States. p. Medium: ED; Size: 5.99 MB.
43. Vena. *Industry Benchmarks of Gross, Net and Operating Profit Margins*. 2025 [cited 2024 4/28/2025]; Available from: https://www.venasolutions.com/blog/average-profit-margin-by-industry.
44. Handyman Blog. *How Much Does Window Replacement Cost?* 2025 [cited 2025 4/28/2025]; Available from: https://www.mrhandyman.com/blog/how-much-does-window-replacement-cost/#:~:text=Keep%20in%20mind%20this%20is,%24200%2D%241%2C200%20per%20window.
45. Homeguide. *Window Frame Repair Cost*. 2023 [cited 2025 4/28/2025]; Available from: https://homeguide.com/costs/window-frame-repair-cost.
46. USGS, *Mineral Commodity Summaries 2021*. 2021.
47. Business Analytiq. *Float glass price index*. 2024 [cited 2024 03/15/2024]; Available from: https://businessanalytiq.com/procurementanalytics/index/glass-price-index/.
48. Made-in-China. *Glass wool price*. 2025 [cited 2025 05/04/2025]; Available from: https://www.made-in-china.com/products-search/hot-china-products/Glass_Wool_Insulation_Price.html.
49. Ibis World. *Price of cement*. 2025 [cited 2025 05/04/2025]; Available from: https://www.ibisworld.com/united-states/bed/price-of-cement/190/.
50. EPA. *Science Inventory - Updated US and Canadian Normalization Factors for TRACI 2.1*. 2014 [cited 2022 01/14/2022]; Available from: https://cfpub.epa.gov/si/si_public_record_Report.cfm?Lab=NRMRL&dirEntryId=275883.
